# AMS-dependent and independent regulation of anther transcriptome and comparison with those affected by other *Arabidopsis *anther genes

**DOI:** 10.1186/1471-2229-12-23

**Published:** 2012-02-15

**Authors:** Xuan Ma, Baomin Feng, Hong Ma

**Affiliations:** 1 Department of Biology and the Huck Institutes of the Life Sciences, the Pennsylvania State University, University Park, PA 16802, USA; 2 Intercollege Graduate Program of Cell and Developmental Biology, the Huck Institutes of the Life Sciences, the Pennsylvania State University, University Park, PA 16802, USA; 3 State Key Laboratory of Genetic Engineering, Institute of Plant Biology, Center for Evolutionary Biology, School of Life Sciences, Institutes of Biomedical Sciences, Fudan University, Shanghai 200433, China; 4 Plant and Microbial Biology Department, University of California, Berkeley, CA 94720, USA

## Abstract

**Background:**

In flowering plants, the development of male reproductive organs is controlled precisely to achieve successful fertilization and reproduction. Despite the increasing knowledge of genes that contribute to anther development, the regulatory mechanisms controlling this process are still unclear.

**Results:**

In this study, we analyzed the transcriptome profiles of early anthers of sterile mutants *aborted microspores *(*ams*) and found that 1,368 genes were differentially expressed in *ams *compared to wild type anthers, affecting metabolism, transportation, ubiquitination and stress response. Moreover, the lack of significant enrichment of potential AMS binding sites (E-box) in the promoters of differentially expressed genes suggests both direct and indirect regulation for AMS-dependent regulation of anther transcriptome involving other transcription factors. Combining *ams *transcriptome profiles with those of two other sterile mutants, *spl/nzz *and *ems1/exs*, expression of 3,058 genes were altered in at least one mutant. Our investigation of expression patterns of major transcription factor families, such as *bHLH, MYB *and *MADS*, suggested that some closely related homologs of known anther developmental genes might also have similar functions. Additionally, comparison of expression levels of genes in different organs suggested that anther-preferential genes could play important roles in anther development.

**Conclusion:**

Analysis of *ams *anther transcriptome and its comparison with those of *spl/nzz *and *ems1/exs *anthers uncovered overlapping and distinct sets of regulated genes, including those encoding transcription factors and other proteins. These results support an expanded regulatory network for early anther development, providing a series of hypotheses for future experimentation.

## Background

In flowering plants, male reproductive organs are called stamens, each of which consists of a filament and an anther [1]. Cells in the anther undergo meiosis to produce microspores, which further develop into mature pollen grains [2]. Therefore, anther development is critical to achieve pollen formation and subsequent success of fertilization [3-6]. According to morphological features, anther development can be grouped into two phases and then be further divided into 14 anther stages [5,7,8]. At the beginning of phase 1 (anther stages 1 to 8), the stamen primordium has 3 layers, L1-L3 from surface to interior. The L1 cells later become the epidermis and the L3 cells give rise to the vascular and connective tissues. Some of the L2 cells develop into archesporial cells which then divide into parietal cells and primary sporogenous cells. Additional cell division and differentiation in the L2-lineage establish a characteristic four-lobed structure at anther stage 5. Each lobe consists of central pollen mother cells surrounded by outer endothecium, middle layer and inner tapetum. Pollen mother cells undergo meiosis at stage 5-6, producing tetrads at stage 7. Dissolution of the tetrad callose wall releases microspores at stage 8. In phase 2, the microspores undergo mitosis and develop into mature pollen grains during stages 9-12. Meanwhile, pollen wall materials are deposited from both the microspores and the tapetum layer. After the degeneration of tapetum, the mature pollen is released and is able to start pollination.

Previous studies indicated that early anther development depends on transcriptional regulation and cell-cell communication [5,7-9]. The *SPOROCYTELESS *(*SPL*)/*NOZZLE *(*NZZ*) gene is one of the earliest genes that regulate anther cell fate determination [10,11]. *SPL/NZZ *is activated by *AG*, a C function gene in the ABC model [12-14]. *SPL/NZZ *is expressed as early as anther stage 2-5 and a mutation in *SPL/NZZ *leads to the failure of differentiation of parietal and sporogenous cells, and consequentially blocks the formation of anther wall and microsporocytes [15,16].

*EXCESS MALE SPOROCYTES1 *(*EMS1*) and *TAPETUM DETERMINANT1 *(*TPD1*) are also essential for male fertility with a later expression peak at stage 5 [17]. EMS1 is a leucine-rich repeat receptor-like protein kinase (LRR-RLKs) and TPD1 is likely its ligand [15,18,19]. In both *ems1 *and *tpd1 *mutants, anthers produce more microsporocytes at the expense of the tapetum, indicating that communication between adjacent cell layers determines the cell fate of archesporial cell progenies in order to form normal anther wall [17]. Besides *EMS1 *and *TPD1*, other cell-cell communication-related genes are also involved in anther development, such as SOMATIC EMBRYOGENESIS RECEPTORLIKE KINASES1/2 (*SERK1/2*), and *RECEPTORLIKE PROTEIN KINASE2 *(*RPK2*) [20,21].

Upon the formation of the anther lobes, *DYSFUNCTIONAL TAPETUM1 *(*DYT1*) and *AMS*, encoding two bHLH transcription factors, are required for tapetal functions at subsequent stages [22,23]. In *dyt1*, tapetum cells harbor enlarged vacuoles and reduced cytoplasm. The *dyt1 *meiocytes have comparatively thin callose walls, cannot complete cytokinesis and finally collapse. RNA *in situ *hybridization experiments showed that *DYT1 *reaches its peak expression at anther stage 5 to 6 [22]. *AMS *functions near the time of meiosis, slightly later than that of *DYT1*. In the *ams *mutant, the microsporocytes can complete meiosis but the tapetum cells prematurely collapse and microspores are degraded before the first pollen mitosis [23]. Beside these regulators, a large number of other genes are also expressed in the anther, and mutations in some of them lead to male sterility by affecting early anther cell formation, tapetum formation, meiosis or pollen maturation [5,7,16,24-28].

However, due to the functional redundancy of members of many gene families, the subtleties of the phenotypes of single-gene mutants, and possible early phenotypes that obscure anther function, forward genetics has limitations in uncovering anther gene functions [29]. Expression profiling has become increasingly informative and might circumvent the limitation of forward genetics. In recent years, global gene expression profiling by microarray has been used to detect floral gene expression and obtain clues for understanding reproductive development. However, most studies to investigate stamen expression profiles have been conducted by analyzing transcripts from the whole inflorescences of male sterile mutants [30-35], rather than the anther itself [32]. Little transcriptomic information about specific organs is currently available, especially for *Arabidopsis *whose male reproductive organs are quite tiny [32,33,36]. Thus the detection of anther-specific or preferential genes in mixed floral tissues might be hampered by the moderate detection sensitivity of microarray technology. As mentioned above, *SPL, EMS1 *and *AMS *have important functions at different stages of anther development, although they have temporal overlap of expression [10,17,22,23]. Therefore, analysis of their shared and distinct effects on the anther transcriptome can shed some light on gene regulatory networks [37-39].

To obtain more information on transcriptomes near the stage of meiosis, we collected anthers at stage 4 to 7 from *ams *mutants and wild-type *Arabidopsis*, even though it is time consuming and technically difficult to dissect developing anthers, because we wanted to identify the genes affected by the *ams *mutation that might be too diluted to detect using RNAs from whole-inflorescences. The *ams *transcriptome data and comparison with previous data from *spl *and *ems1 *anthers [32] provide detailed information on early anther development. Additionally, with known information of other floral organs in *Arabidopsis*, we identified genes that function during early anther stage around meiosis. We found that many transcription factor genes were preferentially expressed during early anther development, such as *bHLH, MYB*, and *MADS*. Closely related homologs were hypothesized to have either redundant or divergent functions according to phylogenic studies [40-42]. Moreover, further investigation of organ-specific transcriptome revealed the importance of both anther-specific and non-specific transcription factors in early anther development. We propose an expanded gene regulatory network that contributes to the precise regulation of temporal and spatial events during early anther development.

## Results and discussion

### Identification of genes regulated by AMS

**Figure 1:**
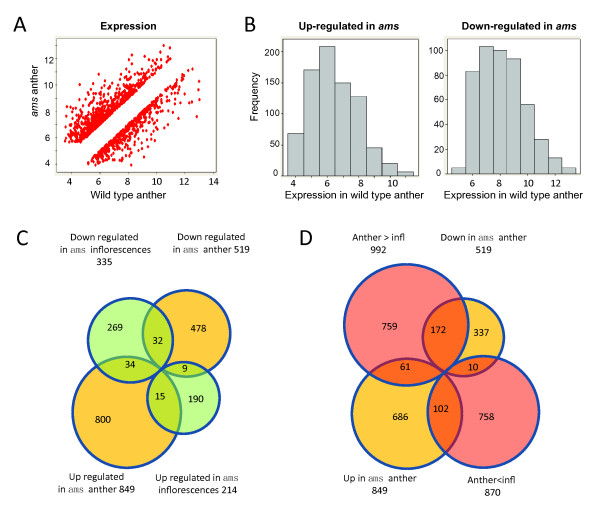
**The expression of genes differentially expressed in *ams *anthers**. (A) A comparison between transcriptome data from *ams *and wild type anthers. All expression data were converted to logarithm base 2 ratio. (B) A histogram of genes with elevated or reduced expression levels between *ams *and wild type anthers. The y-axis the frequency of expression and the x-axis is the log 2 ratio of expression signals. (C) A comparison between *ams *transcriptome data from anthers and inflorescences. (D) A comparison between differentially expressed genes in *ams *anthers and those in wild type inflorescences compared with wild type anthers.

To characterize genes involved in tapetum development and function near the time of meiosis, we isolated total RNA of stage 4-7 anthers from wild-type and the *ams *mutant plants for Affymatrix ATH1 microarray analysis. We included three biological replicates for each genotype and the results are highly reproducible (with correlation coefficients higher than 0.96, Supplemental figure 1 in Additional file 1). We identified 1,368 genes that were differentially expressed in *ams *compared with wild-type anthers with at least 2-fold differences (P < 0.05) (Additional file 2) [32,43]. The scatter-plot of the 1,368 genes shows that they include genes expressed at different levels (Figure 1A,B, 1^st ^sheet in Additional file 2); furthermore, genes with higher expression in *ams *than wild-type tend to have low wild type expression, whereas those with lower than normal expression in *ams *tend to be expressed at higher levels (Figure 1B).

Recently, Xu et al. reported totally 549 genes that are differentially expressed in *ams *floral buds compared with wild-type buds, at four different stages using two color arrays, including 134 genes that were differentially expressed near the time of meiosis (Additional file 2) [35]. Among the 1,368 genes identified in our study, 90 were also identified by Xu et al. in floral buds (Figure 1C). Because *AMS *is expressed from near anther stage 6 (meiosis) through the formation of microspores, our samples from early stage anthers allowed an examination of the early AMS function in regulating transcriptome and sensitive detection of expression shifts without dilution by other floral tissues, resulting in the identification of additional 1,278 genes (478 down- and 800 up-regulated in the *ams *anthers) with differential expression between wild-type and *ams *anthers (Figure 1C).

Nevertheless, our results and the previous study did both detect 90 genes that are significantly affected by the *ams *mutation (Additional file 2; Figure 1C) [35]. Some of these genes show the same direction in expression shifts between the two studies; however, others had the opposite directions (Figure 1C). Specifically, 34 genes with higher expression in the *ams *anther than the wild-type anther had reduced expression levels in the *ams *inflorescence compared with the wild-type inflorescence (Additional file 2); 9 genes showed the opposite trend. These differences might be due to the difference of sampling anther vs. flower bud that included later stages, although other possibilities cannot be ruled out. We observed more similar expression pattern between our anther transcriptome and the published flower bud transcriptome at meiosis stage (Additional file 2). 172 out of the 519 genes down-regulated in *ams *were expressed significantly higher in the wild type anther than the inflorescence, while 102 of the 849 up-regulated genes showed this pattern (P-value < 0.05, Figure 1D, Additional file 2), suggesting that preferential anther expression contributed to the difference between the two studies. It is also possible that the loss of *AMS *function might affect other aspects of flower development than anther development, although not revealed by phenotypic changes.

**Figure 2:**
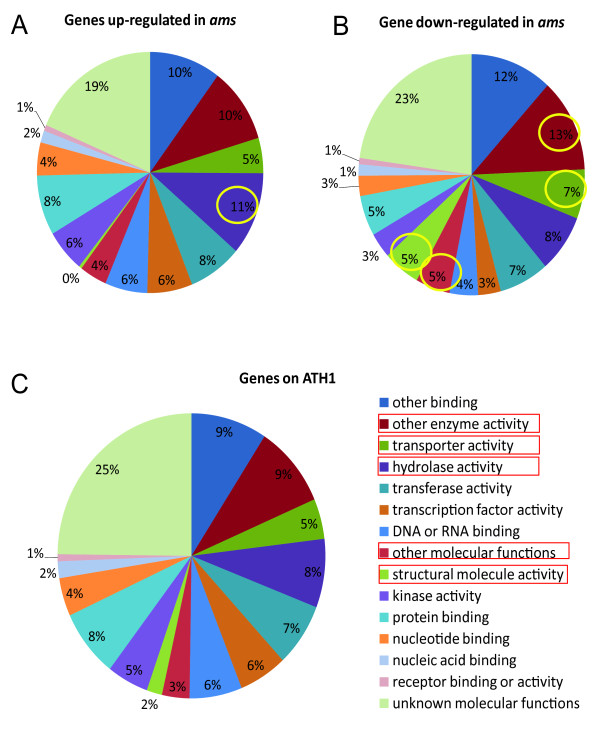
**A pie graph of GO categorization of genes differentially expressed in the *ams *mutant**. (A-B) GO categorization of genes up- and down-regulated in *ams*, with enriched categories circled compared with all genes on ATH1. (C) GO categorization of all genes on the ATH1 chip.

The GO categorization analysis of our anther transcriptome results showed that categories of enzymes, transporters, structural and other molecular proteins were over-represented in the genes with reduced expression, and hydrolases in those with elevated expression levels in *ams *compared with wild-type (Figure 2A-C, Additional file 3). To further investigate the putative functions of genes with different expression patterns in the *ams *anther from inflorescence, we then applied GO categorization to all the newly found differentially expressed genes in the *ams *anther. We found that some categories were enriched in those with reduced expression levels in the *ams *anther, such as structural molecules, transporters, oxidoreductases (supplemental figure 2 & 3 in Additional file 1). These categories are associated with metabolic activities that are very dynamic in tapetum, suggesting a positive role of AMS in regulating metabolic functions in the tapetum. Meanwhile, genes with ion binding, glycosyl-transferase and hydrolase activities were enriched among the genes activated in *ams*.

**Figure 3:**
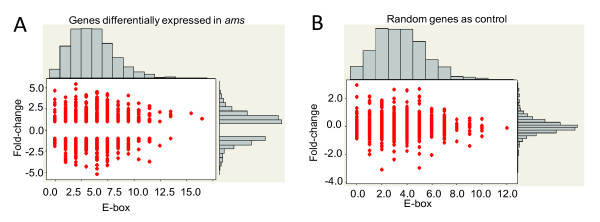
**Marginal plot of fold changes of expression and number of E-box**. (A) Genes differentially expressed in the *ams *anther compared with wild-type anther were selected (more than two fold changes with *P *< 0.05). The logarithm 2 ratio of fold-changes and the number of AMS binding sites (E-box) within 1 kb putative promoter sequence upstream of the start codon were plotted. (B) Randomly selected genes were plotted as control.

As a putative bHLH transcription factor, AMS has the ability to bind to the canonical bHLH binding site (E-box: CANNTG) *in vitro *and *in vivo *[35]. In order to find candidate AMS target genes, we searched E-box elements within 1 kb upstream sequences of genes with statistically significant differential expression between *ams *and wild-type anthers (Figure 3A &3B). We did not find statistically significant interaction between the number of E-boxes in the putative promoter regions and the fold change in gene expression (compared with randomly selected genes on the chip). It is possible that active AMS binding sites are located not just in the 1-kb regions being analyzed, but also in regions further upstream or even downstream of the coding region. It is also possible that a number of the genes affected in the *ams *anthers are indirectly regulated by AMS, hence not containing AMS-binding sites in their promoters.

### AMS affects genes with putative functions in phosphorylation, exocytosis, stress-response and ubiquitin-proteasome pathways during male reproduction

Both somatic and reproductive cells were evidently affected in the *ams *mutant anther, morphologically and transcriptomically [23,35]. Specifically, the *ams *inflorescence showed reduced expression of genes predicted to be involved in metabolism, such as lipid synthesis-related genes [35]. Our anther transcriptome data provided spatially more specific information for the expression patterns of metabolism-related genes (Supplemental figure 4 in Additional file 1) and showed that the expression levels of genes involved in cell wall formation, lipid synthesis and secondary metabolism were obviously altered in the *ams *anther, consistent with morphological defects.

Interestingly, 32 genes located on chloroplast DNA were reduced in expression in the *ams *anthers whereas starch and sucrose related genes were increased (Additional file 4, supplemental figure 4 in Additional file 1). In addition, more metabolism-related genes were found with shifted expression, especially glycosyl-transferase (*P *< 0.01, supplemental figure 5 in Additional file 1,). Besides, the expression levels of genes with putative regulatory functions were also changed, such as kinases and transcription factors (supplemental figure 6-8 in Additional file 1). Interestingly, most of the genes encoding kinases with expression shifts were activated in the *ams *mutant, suggesting a putative negative regulatory role of AMS (supplemental figure 4 in Additional file 1).

In addition, we found that genes likely involved in vesicular transport were up-regulated in *ams*, including genes encoding two SNARE proteins and others related to this process: 3 syntaxins, 3 myosin heavy chains and 2 clathrin proteins (Additional file 4, supplemental figure 9 in Additional file 1). Intracellular trafficking machinery such as SNARE complex is important in animal and plant development [44,45]; for example, one SNARE protein, SEC22, is preferentially expressed in the flower and essential for gametophyte development [46]. Other vesicular transport genes, such as *AtVAM3 *encoding a syntaxin-related protein, were shown to function in vacuolar assembly in *Arabidopsis *[47]. It is possible that the higher than normal expression of genes for vesicular transport contributes to the abnormally vacuolated tapetal cells observed in the *ams *anther [23].

We also found that the expression levels of stress-responsive genes were changed in *ams *(Additional file 4, Supplemental figure 10 in Additional file 1), especially the increased expression of 10 disease resistance genes and two genes encoding respiratory burst oxidases. These findings are consistent with recent studies showing that multiple abiotic stresses can lead to male sterility, such as extreme temperatures and drought [48-50]. In addition, some stress-inducible and/or hormone-related genes were also found with expression alteration, including *RD22*, an ABA-inducible gene responsive to dehydration; *VSP1*, a JA-inducible gene; *EPS1*, a gene possibly acting upstream of SA; *CCR1*, a cold inducible gene; four disease resistance genes encoding TIR-NBS-LRR class proteins; and three heat-shock genes, suggesting complex interactions between internal and external signals regulating anther development and/or functions [51].

Another regulatory pathway activated in *ams *is the ubiquitin-proteasome pathway (Additional file 4), with increased expression of genes encoding subunits of the E3 ubiquitin ligases (Supplemental figure 11 in Additional file 1) [52]. Previous studies demonstrated essential roles of the ubiquitin-proteasome pathway in embryogenesis, hormone signaling, light response, floral development, self-incompatibility, and senescence [48,52,53]. Our results suggested that this pathway may also be regulated by AMS. It is possible that AMS directly regulates the expression of some genes in the ubiquitin-proteasome pathway; alternatively, AMS could influence the expression of such genes indirectly either via AMS-target genes or possibly through the accumulation of damaged proteins which then induce the ubiquitin-proteasome pathway [54]. Further experiments are needed to test these hypotheses.

### Anther-specific or preferential genes were over-represented among genes differentially expressed in the *ams *mutant

Differential expression patterns in vegetative and floral organs can provide clues about gene functions [43]. To find out the relationship between the gene expression shifts in the *ams *mutant and their expression preferences in different organs, we compared our data from wild-type anther with previous microarray data from roots, stems, leaves, seedlings, siliques and inflorescences. The same RNA extraction method and ATH1 platform were applied in both studies so the datasets should be comparable [43]. We defined as anther-specific (A-S) using these criteria: 1) the expression in anther is significantly higher than in any other tissue (with FDR < 0.05); 2) the expression is present in anther but not in any other tissues according to two alternative methods (see materials and methods for details and explanations) [43]. Using the presence call of the MAS5 algorithm identified 124 A-S genes, 76 of which had at least two fold difference; using expression level of 50 as threshold identified 172 A-S genes, 146 of which had at least two fold difference (those with two fold differences are marked with "*" in the second column in Additional file 5). Because both methods for calling "presence" have limitations, only the 43 genes detected by both methods were discussed as A-S gene (this rule also applied to the two groups described below).

Genes were defined as anther-preferential (A-P) if the expression in the anther is: 1) significantly higher than those in any other tissue with FDR < 0.05 (genes with more than 2 fold changes were marked with "*" in Additional file 5 and Additional file 3) present in anther according to the MAS5 algorithm or with expression level of at least 50. Therefore, A-P genes included A-S genes. In addition, those with statistically significantly higher expression levels in anther than in non-floral organs were called reproductive preferential (R-P) genes (Additional file 5, see material and methods for detail). We performed real-time PCR for 6 of these genes and the results were consistent (supplemental figure 12 in Additional file 1). In our result, 24 genes involved in male reproductive development were detected (Table 1). Consistent with previous studies, *SPL *was found in the A-P group and *EMS1 *in R-P group, while *AMS *as an A-S gene [10,17].

**Table 1:** Expression of genes known as anther development related genes

AGI	Name	wt	*s/w*	*e/w*	*a/w*	Function	References

A-S							

AT2G16910	AMS	7.7	-3.4	-3.5	1.7	tapetum dev.	Sorensen et al., 2003

AT1G66170	MMD1	5.5	-1.8	-1.8	-0.1	male meiosis	Alves-Ferreira et al., 2007

AT1G01280	CYP703A2	9.8	-6.2	-6.2	-0.6	pollen dev. and sporopollenin biosynthesis	Souza et al., 2009
		
AT1G62940	ACOS5	11.6	-6.4	-5.7	-1.0		

AT3G11980	MS2	10.6	-6.1	-5.9	-0.6		

AT4G28395	A7	9.6	-4.1	-3.9	0.5		Rubinelli et al., 1998

A-P							

AT2G17950	WUSCHEL 1	7.7	-2.5	0.4	0.5	floral dev.	Ming et al., 2009

AT4G27330	NZZ/SPL	9.3	-3.9	-0.5	-0.5	early anther formation	Ito et al., 2004
		
AT5G14070	ROXY2	9.1	-3.2	-1.7	-0.2		Xing et al., 2008
		
AT3G11440	MYB65	8.9	-2.2	-0.5	-0.4		Millar et al., 2005
		
AT5G06100	MYB33	7.6	-1.1	0.1	-0.4		

AT3G42960	ATA1	11.9	-7.4	-6.7	-1.7	tapetum function	Lebel-Hardenack et al., 1997
		
AT3G51590	LTP12	10.4	-6.1	-5.2	0.9		Ariizumi et al., 2002
		
AT3G28470	MYB35	8.6	-4.9	-4.5	0.0		Zhu et al., 2008

AT1G69500	CYP704B1	11.5	-7.4	-7.0	-2.0	pollen dev. and sporopollenin biosynthesis	Souza et al., 2009
		
AT4G34850	LAP 5	11.4	-6.3	-5.8	-1.6		Dobritsa et al., 2010
		
AT4G35420	DRL1	11.9	-5.1	-4.6	-1.9		Tang et al., 2009
		
AT5G62080	MTG10	13.0	-7.4	-5.2	-4.2		Xing et al., 2007

AT3G22880	DMC1	10.7	-2.2	-0.1	0.0	male meiosis	Doutriaux et al., 1998

AT3G15400	ATA20	12.1	-6.6	-5.6	0.1	pollen wall	Rubinelli et al., 1998

R-P							

AT5G20240	PI	11.8	0.4	0.0	0.2	whorl specification	Li et al., 2008

AT3G17010	B3	8.7	0.9	0.4	0.3	early anther formation	Gomez-Mena et al., 2005
		
At5G07280	EMS1	10.2	-0.9	-2.9	0.1		Zhao et al., 2002
		
AT1G71830	SERK1	8.0	0.8	0.1	0.1		Albrecht et al., 2005

"wt", wild-type; "s/w", fold change of the *spl *signals compared with wild-type; "e/w", of *ems1*; "a/w", of *ams*. A-S represents anther specific; A-P represents anther preferential (A-S excluded) and R-P represents reproductive preferential (A-S & A-P excluded). All expression values are log2 ratio. "dev" means "development" (Column sequence, abbreviation and the version of annotation are used as in tables and supplemental tables below.)

**Figure 4:**
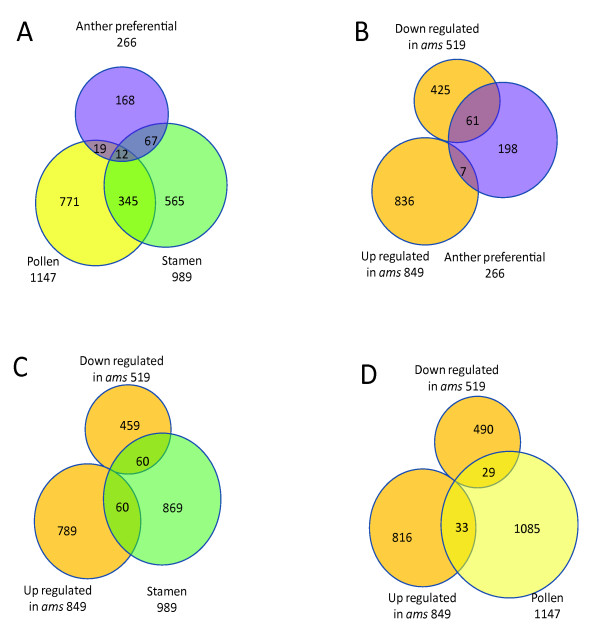
**Venn diagrams of microarray results and previous related study**. (A) A comparison of anther preferential genes identified in our study with previously known pollen genes and stamen genes. (B-D) Comparisons between genes differentially expressed in the *ams *anther and those preferentially expressed in certain organ: anther preferential, stamen and pollen respectively.

Recently, other studies were conducted to identify male reproductive development-related genes. Wellmer et al. identified genes expressed in stamen indirectly by comparing the inflorescence transcriptome of floral homeotic mutants lacking stamens with wild-type [30]. In another study, Honys et al. analyzed microspores/pollen from different stages and defined the male gametophytic transcriptome [33]. A comparison of our A-P genes with these two previous gene lists (defined as stamen and pollen, Additional file 6) revealed that only a small number of genes overlapped between the three male reproductive datasets (Figure 4A, Additional file 6). The differences in identified genes can be explained by the difference of samples used in different studies: our samples only included wild-type anthers at early stages (stage 4-7), whereas the pollen transcriptome data were from microspores and pollen at different stages; and stamen-specific genes was indirectly obtained by subtraction of mutant transcriptome from wild-type and genes in this list might function earlier during organ specification. The dramatic differences between different samples suggest strongly that gene activities alter dramatically between different developmental stages of male reproductive organs [33].

We analyzed the GO categorization for possible enrichment of specific categories among the groups of differentially expressed genes (Additional file 3) and found that, among the 266 A-P genes, the over-represented GO categories were hydrolases, proteins with other binding activities, and other enzymes. No enrichment of other enzyme activity was detected in pollen-specific or stamen-specific datasets found previously [33], suggesting a specific expression profile of early anther development.

Among genes with differential expression in *ams*, the percentage of A-P genes (5%) is significantly higher than its percentage in the whole genome (1%) (Figure 4B, Additional file 2 and Additional file 3). The stamen-specific genes were also enriched among those differentially expressed in *ams *(9%) compared with whole genome data (5%) (Figure 4C &4D, Additional file 2). The results were consistent with our hypothesis that AMS regulates genes with important functions in male-reproductive organ where they have higher expression levels [1,8].

### Genome-wide analysis of gene expression during early anther development by comparing anther transcriptomes of male sterile mutants, *spl, ems1, and ams*

**Figure 5:**
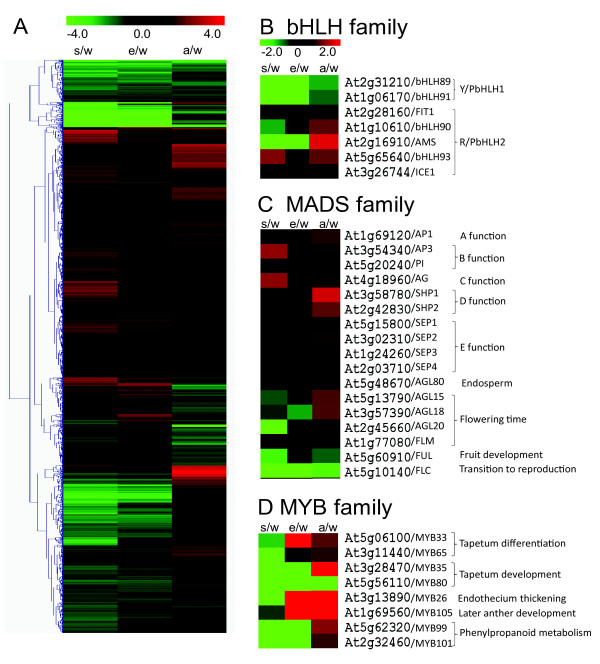
**Expression distribution of all genes differentially expressed and specific gene families**. (A) Hierarchical clustering of genes differentially expressed in at least one mutant. (B-D) Heat-map of *bHLH, MADS, MYB *genes with putative or known function in male-reproductive development. The number indicate logarithm ratio of the fold change in mutant compared with wild-type anther. "w" represents wild-type anther and "s", "e" and "a" represents *spl, ems1 *and *ams*. Red color represents genes which have higher expression level in mutants and green indicates reduced expression.

Previous studies revealed essential roles of *SPL *and *EMS1 *in early anther development and ATH1 microarray data from anthers of these mutants at stage 4-6 were collected and analyzed [32]. To obtain a better overview of early anther development, we analyzed the anther transcriptome data from this study with those of *spl *and *ems1 *(detailed methods applied to all microarray data is described in experimental procedures). 1,813 and 802 genes were identified as differentially expressed in *spl *and *ems1*, respectively, contributing to a total of 3,058 genes that were differentially expressed by 2-fold or more between the wild-type anther and one or more of the *spl, ems1 *and *ams *mutant anthers (Additional file 7). Using the log_2 _values of the ratio of expression of the differentially expressed genes, hierarchical clustering was carried out to obtain heat-maps (Figure 5A). The patterns of *spl *and *ems1 *were similar whereas *ams *had a different pattern, consistent with the fact that the tapetum layer is absent in both *spl *and *ems1 *but is formed in the *ams *anther.

**Figure 6:**
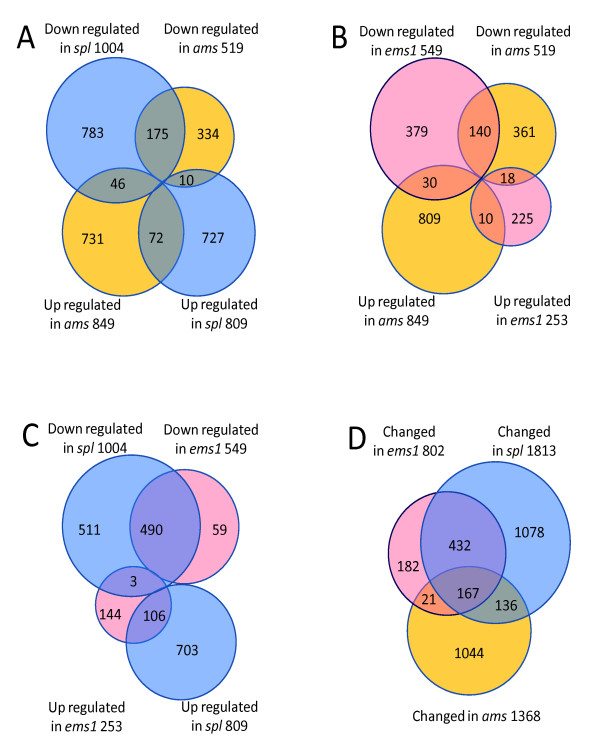
**Comparisons between transcriptome information from three mutants**. (A-C) Genes differentially expressed in three mutants respectively compared with wild-type anther have been identified and then compared with each other pairwisely. (D) Comparison of genes differentially expressed in three mutants compared with wild-type.

In addition, we compared the direction of differential gene expression by pair-wise comparison between different mutants, as shown in Venn diagrams (Figure 6A-D) and found that many more genes showed changes in the same direction in all three mutants than genes with changes in the opposite direction, suggesting that the three transcription factors had similar effects on some of the target genes. We also found that the non-overlapping (differentially expressed in one mutant, but not in either of the other two) percentage of differentially expressed genes in *ams *(76%) is larger than those in *spl *and *ems1 *(59% & 23%, respectively, Figure 6D), providing strong evidence at the transcriptome level that the AMS function was distinct from those of SPL and EMS1 and likely regulates late gene expression in anther development, consistent with other studies [23].

Because the three mutants showed related but distinct phenotypes, we speculate that the functions of genes differentially expressed in these mutants might differ from each other. Thus we applied GO categorization of molecular function to genes up- or down-regulated in each mutant (in Additional file 3). First, genes annotated to have "other binding activities" and "other enzyme activities" were significantly enriched in categories with reduced expression in each mutant (*P*-value < 0.05), consistent with previous knowledge of dynamic metabolism in tapetum cells. In addition, genes encoding transcription factors and DNA binding proteins are enriched in categories with both up-and down-regulated genes in the *spl *mutant, suggesting that SPL control anther development at least in part by regulating genes encoding transcription factors. Furthermore, the *ams *mutant showed reduced expression of many genes encoding structural proteins, which mainly contribute to cell structural integrity, suggesting that AMS might activate these genes to promote maturation of tapetum cells.

### SPL and EMS1 might control tapetum development by activating AMS-dependent gene expression

To gain a better understanding of genes that may function together in anther development, we divided the 3,058 genes into different clusters based on their expression patterns. Totally 136 genes had repressed expression in all three mutants (Additional file 7). Since tapetum cells are either absent or dysfunctional in the mutants, we expected that the expression of tapetum-related genes would reduce significantly. Previous studies indicated that tapetal cells were primarily involved in nutrition and material provision for pollen maturation [2]. Consistent with this notion, genes encoding enzymes in this group (21.8%) are obviously over-represented comparing with all genes on ATH1 chip (10.1%). Besides, genes belonging to the other binding category were also enriched in this group (16.4% V.s 9.9%, Additional file 3).

Among these genes, many of them are involved in biosynthesis of pollen wall-related compounds, such as lipids, lignin and flavonoids. A recent study showed that the loss of acyl-CoA synthetase, GhACS1 , which might be involved in biosynthesis and transfer of lipids, can lead to male sterility in cotton [55]. The expression levels of 8 *Arabidopsis *genes involved in the lipid metabolism pathway were significantly reduced in the three mutants, suggesting their potential roles in metabolism in tapetal cells (Table 2). Besides, we found that the expression levels of 30 genes involved in endomembrane system decreased in all mutants (Table 3). Recent studies in plants suggested that many endomembrane proteins might be involved in trafficking thus influencing signal transduction and development [56-58]. Based on the observation of tapetum defects in all three mutants [10,17,22], we speculate that genes sharing similar expression patterns might be important for maintaining the tapetum identity.

**Table 2:** Genes significantly down-regulated in all three mutants are involved in metabolism of pollen wall formation, including lipid, pectin, lignin and exine

AGI	wt	*s/w*	*e/w*	*a/w*	Function	Expression
lipid related						

At5g61320	8.7	-3.9	-3.6	-2.2	cytochrome P450 - like protein	A-S
		
At5g08250	8.9	-4.5	-4.1	-1.4		A-P
	
At1g06250	7.6	-3.2	-3.0	-1.8	lipase-like protein	
	
At5g62080	13	-7.4	-5.2	-4.2	lipid-transfer protein	
		
At3g07450	12.8	-7.5	-5.6	-5.2		
		
At3g52130	12.9	-7.4	-6.0	-3.3		
		
At5g07230	10.2	-8.1	-6.5	-2.7		
		
At5g52160	10.7	-6.3	-6.2	-1.7		

pectin						

At3g24230	7.6	-3.2	-3.2	-3.2	pectate lyase	A-P
		
At4g22080	8.5	-4.9	-4.6	-1.5		
	
At1g75790	10.2	-5.8	-5.5	-1.9	pectinesterase like protein	

At3g01270	6.2	-1.2	-1.2	-1.1	putative pectate lyase	R-P
	
At5g50030	6.5	-1.3	-1.1	-1.4	pectin methylesterase inhibitor	

lignin						

At1g76470	10.3	-4.8	-5	-2.3	putative cinnamoyl-CoA reductase	A-P

At3g21230	7.7	-3.1	-3.1	-1.8	4-coumarate-CoA ligase 2	

exine						

At3g13220	10	-5.9	-5.7	-2.2	WBC27 white-brown complex	A-S

At4g14080	12.5	-8.1	-7.1	-3.8	maternal effect embryo arrest 48	A-P
	
At1g02050	11.6	-4.6	-4.7	-1.9	LESS ADHESIVE POLLEN 6 (LAP6)	

All the expression values are log2 ratio.

**Table 3:** Genes related to endomembrane system affected by SPL, EMS1 and AMS

AGI	wt	*s/w*	*e/w*	*a/w*	Function	Expression
At5g24820	10	-5.7	-5.4	-2.1	cnd41	A-S
	
At3g23770	9.9	-5.2	-5.3	-2.7	beta-1,3-glucanase, putative	
	
At1g28375	9.7	-5.4	-5.1	-2.3		
	
At3g21620	7.2	-2.7	-2.5	-2.1		
	
At4g30040	6.6	-2.0	-2.1	-1.1	cnd41	

At2g24800	8.1	-4.2	-4.2	-2.3	peroxidase	A-P
	
At1g61070	10.2	-6.1	-5.7	-1.1	defensin	
	
At4g14080	12.5	-8.1	-7.1	-3.8	A6 anther-specific protein	
	
At1g75790	10.2	-5.8	-5.5	-1.9	pectinesterase like protein	
	
At4g20420	11.7	-6.0	-5.4	-3.1	tapetum-specific A3	
	
At1g76470	10.3	-4.8	-5.0	-2.3	cinnamoyl-CoA reductase	
	
At2g21430	10.9	-4.5	-4.1	-2.1	cysteine proteinase	
	
At1g02640	11.4	-2.0	-2.1	-1.2	beta-xylosidase	
	
At1g04645	9.6	-5.3	-3.3	-3.4		
	
At2g15120	8.2	-4.1	-3.4	-4.3		
	
At4g20050	9.9	-5.3	-5.0	-1.6		
	
At5g04820	8.8	-1.6	-2.2	-1.2		
	
At4g29980	11.5	-5.9	-5.1	-4.1		
	
At1g22015	10.0	-4.1	-3.8	-2.7		
	
At1g28710	9.4	-4.0	-4.1	-2.3		

At5g18290	9.2	-2.3	-2.3	-1.7	SIP1	R-P
	
At4g32105	8.1	-2.4	-2.5	-2.3		
	
At1g49490	6.6	-1.7	-1.9	-2.0		
	
At1g32170	7.4	-1.1	-1.3	-1.2	endoxyloglucan transferase	
	
At4g16563	6.9	-1.4	-1.0	-1.2	nucleoid DNA-binding	
	
At5g50030	6.5	-1.3	-1.1	-1.4	pollen-specific protein	
	
At5g45880	7.1	-1.3	-1.0	-1.3	Ole e I	
	
At5g44380	5.9	-1.2	-1.2	-1.7	reticuline oxidase precursor	
	
At5g14300	5.8	-1.4	-1.3	-1.3	prohibitin - like protein	
	
At5g12940	8.3	-2.0	-2.1	-1.1	leucine rich repeat protein	
	
At5g09520	5.9	-1.9	-2.0	-1.8	surface protein PspC-related	
	
At3g06300	9.8	-2.1	-1.3	-1.1	4-hydroxylase alpha subunit	
	
At1g60390	9.4	-2.0	-2.3	-1.2	polygalacturonase isoenzyme	
	
At3g05930	6.7	-1.4	-1.3	-1.2	germin-like protein	
	
At1g02790	6.2	-2.0	-1.7	-1.5	polygalacturonase	
	
At5g09730	8.2	-3.8	-2.7	-1.9	beta-xylosidase	
	
At5g51950	8.2	-4.2	-4.1	-1.3	mandelonitrile lyase	
	
At1g30760	7.4	-3.5	-3.3	-3.1	reticuline oxidase-like protein	
	
At1g22890	7.7	-2.9	-1.1	-1.9		
	
At1g33055	7.8	-1.9	-1.9	-2.5		
	
At1g49500	9.3	-1.4	-1.5	-1.7		
	
At3g22640	7.6	-3.0	-1.6	-3.1		
	
At4g15750	6.4	-1.2	-1.2	-2.1		

All the expression values are log2 ratio.

In addition, five genes for potential transcription factors were also found in this category (Table 4). Among them, At5g58610 and *AGL25*/At5g10140 are A-P genes. At5g58610 has a putative function in pathogen defense reaction, uncovering a possible factor in both anther development and external biotic stress response pathways [59,60]. *AGL25*, also known as *FLC*, is a repressor of flowering and its expression is epigenetically regulated [61]. However, its possible function in anther development is not known. Three others were *AGL40*/At4g36590, *MYB80*/At5g56110 and *HAT9*/At2g22800. *AGL40 *was found in the proliferative endosperm transcriptome and *MYB80*/At5g56110 in tapetum development [27]. These results suggested that normal tapetum functions might require multiple transcription factors preferentially expressed in the anther downstream of AMS.

**Table 4:** Transcription factors in SEA-L and SE-L cluster with known or putative function in anther development

Cluster	AGI	wt	*s/w*	*e/w*	*a/w*	Function	Expression
SEA-L	At5g58610	7.7	-1.6	-1	-1.9	PHD finger	A-P
		
	At5g10140	8.1	-3.2	-3.1	-1.8	AGL25	
	
	At4g36590	5.8	-1.7	-1.4	-1.7	AGL40	R-P
	
	At2g22800	8.7	-2.9	-2.0	-1.3	HAT9	
		
	At5g56110	7.5	-1.6	-1.5	-1.8	MYB 80/MYB103	

SE-L	At1g06170	9.6	-5.9	-4.6	-0.8	bHLH89	A-P
		
	At3g28470	8.6	-4.9	-4.5	0.0	TDF1/MYB35	
		
	At2g31210	7.1	-3.4	-2.9	-1.1	bHLH91	
	
	At3g57370	7.3	-2.8	-2.8	0.8	initiation factor IIB	R-P
	
	At1g77850	8.8	-2.4	-1.7	-0.1	auxin response factor	
		
	At2g28830	6.5	-1.1	-1.1	0.3	transcription activator	
		
	At5g62320	8.6	-3.3	-3.4	-0.3	MYB99	
		
	At4g09460	8.7	-3.4	-2.3	0.8	MYB6	
		
	At4g34680	9.3	-2.6	-1.2	0.3	GATA 3	
		
	At2g41630	10.6	-1.1	-1.4	-0.7	TFIIB	
		
	At3g10580	7.3	-2.5	-2.3	0.9	MYB	
		
	At5g61590	9.7	-2.3	-1.6	-0.3	AtERF107	
		
	At4g37790	8.1	-1.9	-1.8	-0.8	HAT22	
		
	At4g34990	8.5	-1.3	-2.2	-0.6	MYB32	
		
	At4g10920	8.0	-1.9	-1.8	-0.5	transcriptional co-activator	
		
	At1g66160	7.0	-2.8	-2.0	-0.5	PHOR1 like	
		
	At5g65790	7.5	-3.7	-3.2	-0.1	MYB68	
		
	At4g34000	7.7	-1.8	-1.4	-0.2	OBF3	

All the expression values are log2 ratio. SEA-L includes genes whose expression levels were reduced in all three mutants. SE- L include genes whose expression levels were reduced in both *spl *and *ems1 *mutants.

### SPL and EMS1 can regulate early anther development by AMS-independent pathways

Moreover, 354 genes showed reduced expression in *spl *and *ems1 *but not in *ams *(Additional file 7), including the enrichment of the categories of hydrolase activity (15.5% vs. 8.4%), other binding activity (19.5% vs. 9.9%), and other enzyme activity (18.0% vs. 10.1%). Among the genes in this cluster, four genes: *MS2, ACOS5, CYP703A2 *and *A7*, were involved in sporopollenin monomer biosynthesis, the lack of which leads to male sterility (Table 1) [62,63]. Since these genes were not affected in the *ams *mutant, some lipid metabolic genes might be activated independent of AMS and they might exert functions earlier than AMS or in parallel to AMS [63,64].

Besides, several genes encoding putative transcription factors were found within this subset (Table 4). A-P genes with known functions, such as *TDF1*/At3g28470 (or *MYB35*) and *bHLH89*/At1g06170, were also identified in this category [24,32,35]. TDF1 is essential to the tapetum function controlling callose dissolution and acts downstream of SPL and upstream of AMS and MYB103 (Table 4) [24]. Our data also support the regulatory hierarchy of SPL-TDF1-AMS.

**Figure 7:**
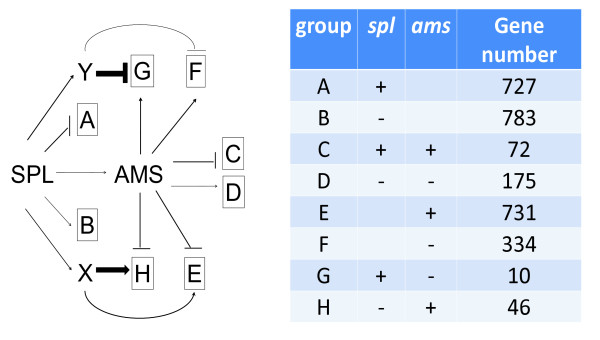
**AMS-dependent or -independent regulatory model during anther development**. Genes with differential expression in the *ams *mutant is divided into eight groups A-H (plus represents for higher expression in mutant compared with wild-type and minus for lower). Negative regulation is shown by a T-bar and positive by an arrow, and stronger impact is shown by a thicker T-bar or arrow.

The expression of *AMS *is significantly reduced in *spl*, therefore we assumed that genes down-regulated in *ams *should have similar reduction in *spl*. Interestingly, we found that 56 genes showed opposite expression changes in *spl *and in *ams *compared with wild type anther, and even larger proportion (1,065 genes) only differentially expressed in *ams *(Figure 6A). Another gene with reduced expression in *spl *and *ems1 *mutants is *DYT1*, which encodes a bHLH protein similar to AMS [22]. It is possible that SPL might also regulate anther development through pathways independent of AMS, such as those requiring DYT1 function [22]. We speculate that SPL might activate other transcription factors that affect AMS-regulated genes in contrast to the function of AMS (represented by factors × and Y in Figure 7). The effects of AMS reduction in *spl *might be outweighed by the loss of × or Y; such regulatory interactions would explain the opposite expression changes in *spl *and *ams*. The identification and understanding of the proposed factors will require further investigations.

### AMS-dependent and independent anther expression of genes encoding transcription factors

#### Expression of bHLH genes during anther development

Since many transcription factors have been found to play key roles in regulating anther development, we analyzed our anther transcriptome profiles by focusing on transcription factor gene families [5,28,65]. To identify additional candidate genes for anther development, we analyzed all 147 known bHLH genes in *Arabidopsis *(Additional file 8) [40]. For several clades according to the most recent phylogeny trees of bHLH family [40,66,67], including the clade that includes AMS, all or most members of the same clade were expressed similarly in the anther (Additional file 8), suggesting conserved functional roles in anther development. For example, bHLH91 and bHLH89 shared similar reductions in all three mutants, suggesting possible redundant functions in the anther (Figure 5B).

In other cases, the closely related homologs did not share similar expression patterns in mutant vs. wild type anthers (Additional file 8). For example, *bHLH93 *is a close homolog of *AMS*; but unlike *AMS*, it was preferentially expressed in the inflorescence compared with the anther. Also unlike *AMS*, it was elevated in expression in *spl*. It is possible that some compensatory mechanisms might act to increase transcription of *bHLH93 *when *AMS *is mutated (Figure 5B).

In addition, some bHLH genes with known functions in other organs showed increased expression in the *spl *mutant, suggesting that *SPL *acts to maintain the identity of male reproductive organ by reducing the expression of genes needed for other organs. For example, *ZCW32 *(*bHLH31*) controls petal formation and was activated in the *spl *anther [68,69], suggesting that SPL can promote the normal anther development at an early stage by repressing some genes normally expressed in nearby whorls.

#### Possible role of MADS-box genes in anther development

Genes of the MADS-box family have been extensively studied in *Arabidopsis*, because they were first identified as flower homeotic genes that determine floral organ and meristem identities [70,71]. Till now, more than one hundred MADS-box genes have been identified, 79 of which were found to be present in our anther microarray data but most were non-anther-specific (Additional file 7 & Additional file 8) [70]. Except for *APETALA2 *(*AP2*), majority of genes involved in the ABCDE model belong to the MADS family [71]. They are mostly inflorescence-preferential rather than anther-specific genes from the comparison of microarray data as described above. *APETALA1 *(*AP1*) is an A function gene controlling the first and second whorls and no expression shift was observed [72]. *APETALA3 *(*AP3*) and *PISTILLATA *(*PI*) are both B function genes, essential for the formation of petals and stamens [72-74]. Interestingly, their expression patterns were different. *PI *is an anther-preferential gene, but its expression level did not change in any mutant while *AP3 *was obviously up-regulated in *spl*, suggesting that *AP3 *is regulated more tightly than *PI *during anther development *. AG*, the C class gene controlling both stamen and carpel identities, shared similar expression patterns in the anther with *AP3 *[13], supporting a role of *AG *in anther development after the specification of stamen identity (Figure 5C).

Moreover, D class genes, including *STK*/AT4g09960, *SHP1/*At3g58780 and *SHP2*/At2g42830 , are important for ovule development [75,76]. Although the expression of D class genes was relatively low, we observed increased expression of *SHP1 *in the *ams *mutant, suggesting a possible negative regulatory role of AMS in ovule development. On the other hand, E class genes, *SEP1, SEP2, SEP3 *and *SEP4*, which are homologs that have redundant functions, had different expression pattern in the anthers. *SEP1 *and *SEP2 *were activated in *spl *and *ams*, whereas *SEP3 *and *SEP4 *did not change much (Figure 5C).

Beside the ABCDE genes, some other MADS genes were also expressed in the anther (Additional file 8). The expression levels of known flowering-time related genes (*FLM, AGL15, AGL18 *and *AGL20*) [77-79] were reduced in *spl *and *ems1 *slightly. *FUL *involved in fruit development [80] was up-regulated in the *spl *and *ems1 *mutants, suggesting negative roles of SPL and EMS1 in whorl 4. *AGL80*, important for central cell and endosperm formation in female gametophytes [81], was also reduced in all three mutants, suggesting a possible role in male gametophyte (Additional file 8).

#### Differential expression of MYB genes in three mutants

In addition to the bHLH and MADS-box families, other gene families are also involved in anther development. As the largest *Arabidopsis *transcription factor family, *MYB *genes play important roles in controlling many cellular processes, such as secondary metabolism, morphogenesis, and signal transduction (Additional file 8) [82]. Previous studies revealed a number of roles of MYB genes in early anther development (Figure 5D). For example, GAMYB in rice functions in anther development via GA signaling pathway [83]. In *Arabidopsis*, the GAMYB homologs *MYB33 *and *MYB65 *also share a redundant function regulating tapetum differentiation [22,27,84,85]. Our microarray results indicated that expression of *MYB33 *and *MYB65 *was reduced only in *spl*, not in the other two mutants, implying that the functions of *MYB33 *and *MYB65 *are independent of EMS1 or AMS.

In addition, *MYB35*/*TDF1 *and *MYB80*/*MYB103 *controlling callose dissolution and exine formation [27] were reduced in *spl *and *ems1*, and *MYB80 *was also down-regulated in *ams*, suggesting that it acts downstream of *AMS*. Moreover, the *MYB99 *and *MYB101 *genes that regulate phenylpropanoid metabolism [31] showed a similar expression pattern to that of *MYB35*/*TDF1. MYB26*/*MS35 *and *MYB105 *are closely related homologs; both were down-regulated in *spl *but up-regulated in *ams*. Previous study suggested that *MYB26 *is required for endothecium thickening and anther dehiscence [86]. RNA *in situ *hybridization revealed that *MYB105 *as well as *MYB101 *are expressed in late tapetum [86,87], consistent with our findings of the changes of their expression in the mutant anthers.

#### Expression of WRKY, bZIP, AP2/ERF and NAC genes

The *WRKY *family contains at least 72 members in *Arabidopsis *[42] and has diverse functions, such as abiotic and biotic stress response, hormone signaling pathway, immune response and development in plants [88]. However, it is not known whether *WRKY *genes are important for flower development. Here we compared the expression of all *WRKY *genes on the ATH1 chip and found that 29 of them were expressed in the anther (Additional file 8), with the highly similar *WRKY2*/At5g56270 and *WRKY32*/At4g30935 [88] being anther-preferential. Moreover, *WRKY2 *was down-regulated in *spl*, suggesting that it might function downstream of *SPL *in anther development.

We also analyzed bZIP, ERF and NAC families of transcription factors. Like the WRKY family, most genes in these families do not have known functions in reproductive development (Additional file 8). However, we found several of them were differentially expressed in the anthers of male sterile mutants, suggesting they are components of a complex transcriptional network regulating anther development.

#### Transcriptional regulatory network for anther development

**Figure 8:**
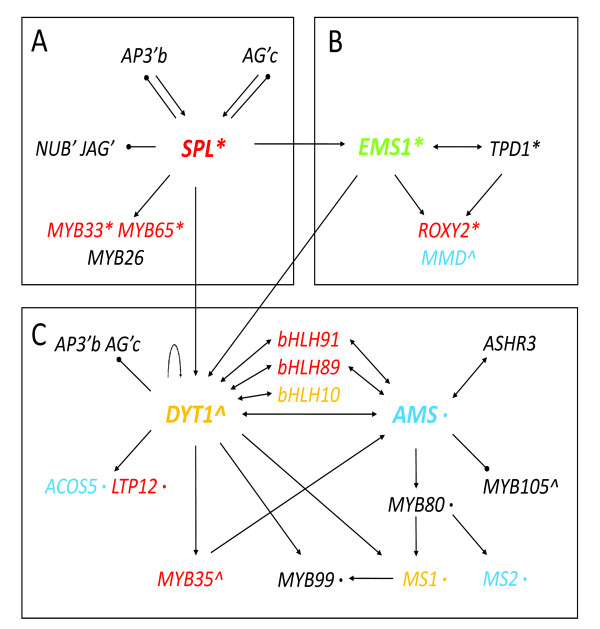
**Gene regulatory network of anther development during early stages**. Gene regulation is represented by T-bars (negatively) and arrows (positively). The direct regulation confirmed by experiment is represented in bold line. Genes encoding proteins with interaction is represented by double arrows. Gene expression patterns in different tissues are shown by colors (blue for anther specific; red for anther-preferential; green for reproductive-preferential and yellow for genes not included in ATH1 chip). Gene function in tapetum formation is marked by an apostrophe; in pollen wall formation by an asterisk; in callose dissolution by double asterisks; in stamen and petal formation by the letter b; in stamen and carpel formation by the letter c.

Genetic studies and our transcriptomic analyses reported here support an emerging transcriptional network (Figure 8). Previous molecular genetic studies showed that SPL up-regulates the expression of *EMS1 *and *DYT1*, which are upstream of *AMS *[22,23], as well as other genes encoding transcription factors shown to be important in anther development [8]. *SPL *also negatively regulates the expression of B and C function genes in the anther, as well as some genes that are normally expressed in petals and carpals, probably to prevent anther from developing traits of other floral organs. In addition, the key position of *SPL *in anther regulatory hierarchy as indicated by genetic studies is supported by its effects on the anther transcriptome (Figure 8A).

EMS1 also positively regulates the expression of *DYT1 *[32]. EMS1 was shown to interact with its putative ligand TPD1 [18], thereby regulating genes essential for the differentiation of tapetum cells. In addition, some genes important for meiosis are also affected in the *ems1 *mutant. For example, the MMD and ROXY2 genes that are important in anther lobe formation and meiosis, respectively, were significantly reduced in *ems1 *(Figure 8B).

*AMS *was down-regulated in *spl *and *ems1 *according to the microarray data. Because DYT1 and AMS are related bHLH proteins, which are known to form homodimers or heterodimers with other bHLH proteins, we propose that they probably regulate the expression of different genes by forming different complex with other proteins. DYT1 is also a putative candidate that exerts opposite function as × and/or Y downstream of SPL in anther development by interact with different proteins (Figure 8C). This proposed transcriptional regulatory network of anther development is based on information from genetics, transcriptomics, and phylogenetics studies (Figure 8A-C). The hypothesized interactions, including the roles of some functionally redundant genes, could be tested by further experiments.

## Conclusion

In this study, we identified genes whose expression was changed in *spl, ems1 *and *ams *at anther stage 4-7 and further categorized these genes according to their expression patterns. These genes might directly regulate some fundamental biological processes during anther development. In addition, both anther-specific and non-anther-specific genes are identified in anther development. Transcriptome analyses also showed AMS-dependent and -independent pathways. Careful analyses of transcriptome combined with genetic and phylogenetic information revealed an elaborate regulatory network during early anther development and expanded our understanding of the hierarchy of anther-development-related genes, especially transcription factors.

## Methods

### Plant materials

All the plants in this study were grown in soil under long day condition (16 h light/8 h dark) at constant 22°C. The wild-type in this paper refers to ecotype Landsberg *erecta *(L *er*). The mutants of *spl, ems1 *are of L *er *background as described [22,32], while the *ams *mutant is of Columbia background. We select 21-28 day old plant to collect anther at 4-7 stage as described previously [32].

### Microarray experiment

Following the Affymetrix GeneChip Expression Analysis Overview described on the website [35], cRNA was synthesized for hybridization as described [32]. Hybridization, washing, staining, scanning and data collection were performed at the Genomics Core Facility, Pennsylvania State University, University Park.

### Microarray analysis to identify differentially expressed genes in anther of mutants

Normalization was applied using Bioconductor package in R by RMA [43], and all expression values were converted to logarithms base 2. LIMMA library was then used to compare signals from mutant and wild-type anther. Only genes with more than two-fold changes were selected. To obtain more reliable result, we screened out genes with q-value (FDR) larger than 0.05, since q-value is more stringent than p-value of *T*-test based on previous study [89].

Similar data processing was performed with the microarray results from different organs. The microarray data from all organs in wild-type *Arabidopsis *were normalized together and converted to logarithms base 2 values. We defined genes as anther-specific if they met these criteria: 1) the expression in anther is significantly higher than in any other tissue with FDR < 0.05; 2) gene is present in anther but absent in any other tissues. We used two alternative methods to define whether a gene is present in a tissue. One of the methods was using the Affymetrix' MAS5 algorithm. This method uses a comparison of hybridization intensity with wild-type oligo set vs mismatched oligo set; sometimes similar levels of hybridization to both sets can actually be real expression, yet such results would lead to "absent" calls. Therefore, we also used a second method to define "presence", by using a threshold of 50 for expression value, previously determined on basis of analysis of variation among samples of the same tissue [32,43]. Both results are shown in Additional file 5.

For the anther-preferential genes, we used the criteria that the expression in anther is 1) present using both MAS and/or 50 cutoff; 2) significantly higher than in any other tissue with FDR < 0.05; 3) at least 2 fold more compared with any other tissues. The reproductive-preferential genes required the expression present and significantly higher in anther than only the vegetative organs using FDR < 0.05 and 2-fold changes.

Hierarchical clustering of co-expressed genes was performed by MeV 4.6 [86]. We used Euclidean distance metric to conduct this analysis. For the identification of the functions of the differentially expressed genes, the annotations of genes on ATH1 microarray chip were downloaded from Affymetrix website and we used the GO categorization function on TAIR website [71]. To verify whether one category is enriched compared with the whole genome, we applied hypergeometric test and only the categories with p-value less than 0.05 were called statistically enriched group [90].

### Cis-regulatory element analysis

Possible promoter sequences of all genes on the microarray chip (1 kb upstream of the start codon) were obtained from TAIR website. The number of common bHLH binding site (E-box) was then counted. We then plotted the fold-changes of gene expression in *ams *against the numbers of their putative AMS binding sites using minitab [47]. The identification of *cis*-regulatory binding site was conducted by perl [46]. The binding motifs were obtained from Gene Regulation and PlantCARE [70].

### Real-time PCR experiments

To test the reliability of our microarray hybridizations, six genes and one reference (ACT2, At3g18780) were studied using Quantitative Real-Time PCR. RNA extraction and Real-Time experiments followed the protocols described previously [91]. Triplicate reactions were performed for all tissues with "no reverse transcription" as a negative control. All primer information is provided in Additional file 5. Relative transcript quantities were calculated using the ΔΔCt method [92].

## Abbreviations

AMS: Aborted microspores; AG: Agamous; AP2/ERF: APETALA2/ethylene response factor domain-containing transcription factor; bHLH: Basic helix-loop-helix; bZIP: Basic-leucine zipper; CCR1: Cinnamoyl coa reductase1; DYT1: Dysfunctional tapetum1; EMS1: Excess male sporocytes1; EPS1: Enhanced pseudomonas susceptibilty; EXO: Exocytosis; GO: Gene ontology consortium; MADS: MCM1-agamous-deficiens-SRF; MYB: Myeloblastosis-like gene; NAC: NAM/ATAF1/2/CUC2; RD22: Responsive to dessication22; PI: Pistillata; RPK2: Receptor-like protein kinase2; SEC: Secretion; SERK1/2: Somatic embryogenesis receptor-like kinases1/2; SNARE: Soluble NSF attachment protein receptor; SPL/NZZ: Sporocyteless/nozzle; TAIR: The arabidopsis information resource web site; TIR-NBS-LRR: Toll interleukine 1receptor-nucleotide binding site-leucine rich repeat domain;TPD1: Tapetum determinant1; VAM3: Vacuolar morphology; VSP1: Vegetative storage protein.

## Authors' contributions

HM designed and supervised the research; BF performed tissue collection and RNA isolation; XM performed data analysis; XM and HM wrote the manuscript drafts; XM, BF and HM edited the manuscript; all authors approved the manuscript.

## Supplementary Material

Additional file 1 **Figure S1. Correlation coefficients between signal intensities from wild-type and the *ams *anther replicates**. Pearson's correlation coefficients were larger than 0.96 between pair of the biological replicates from the *ams *and wild type anther, indicating that the results were highly reproducible. **Figures S2 & S3. GO annotation of genes up- and down-regulated in *ams*. **GO categorization of genes differentially expressed in the *ams *mutant compared with wild type. The enriched groups were shown in different color with P-value provided. **Figures S4-S11. The genes involved in different metabolic pathways that were activated or repressed in *ams *compared with wild type**. Red color represents genes activated while green color represents genes repressed in *ams *compared with wild type. The overview of metabolism activities was shown in supplemental figure 4. Figure 5, 6, 7, 8-11 showed expression shifts of genes involved in secondary metabolism, regulatory pathways, receptor-like-kinase pathway, transcriptional regulation, protein trafficking, stress response, ubiquitin and autophagy dependent degradation pathway. **Figure S12. Real-time PCR results consistent with microarray data**. Six genes were verified using real-time PCR. The bars in blue represent the real-time RT-PCR results while red the microarray results. All the numbers shown in this figure are the fold changes of expression intensities in other tissues compared with anther. "infl" is the abbreviation of inflorescence. Click here for file

Additional file 2 **Genes differentially expressed in anther and inflorescences from the *ams *mutant**. This additional file contains information about genes differentially expressed in the *ams *anther and inflorescences compared with wild type. Column sequence, abbreviation and the version of annotation are as those used as in table 1 and all the other supplemental tables. All expression values are log2 ratio. Click here for file

Additional file 3 **GO categorization of different clusters based on expression pattern**. This additional file contains information about numbers of genes in each GO category. The enriched categories were highlighted in red color. Click here for file

Additional file 4 **Genes differentially expressed in the *ams *mutant with putative function in exocytosis, transportation, ubiquitination and stress reaction**. This additional file contains information about genes involved in different pathways with elevated expression levels in *ams*. Click here for file

Additional file 5 **Genes defined as specifically or preferentially expressed in early anther or preferentially expressed in reproductive tissue**. This additional file contains information about genes preferentially expressed in only anther or reproductive tissues compared with roots, stems, leaves, siliques. Click here for file

Additional file 6 **Genes expressed in stamen, early anther and pollen**. This additional file contains information about the expression levels of gene in different organs. Click here for file

Additional file 7 **Genes differentially expressed in *spl***, ***ems1 *or/and the *ams *mutants**. This additional file contains information about the expression levels of gene differentially expressed in the three mutants. Click here for file

Additional file 8 **Expression pattern of *MADS***, ***MYB***, ***bHLH***, ***WRKY***, ***bZIP***, ***AP2/ERF *and *NAC *families**. This additional file contains information about the expression levels of different gene families. Click here for file
