# Supplementary material for: AMS-dependent and independent regulation of anther transcriptome and comparison with those affected by other Arabidopsis anther genes
Source: BMC Plant Biol. 2012 Feb 15;12:23. doi: 10.1186/1471-2229-12-23 (PMC3305669; doi:10.1186/1471-2229-12-23)
Supplement: Additional file 8 — Expression pattern of MADS, MYB, bHLH, WRKY, bZIP, AP2/ERF and NAC families. This additional file contains information about the expression levels of different gene families. [file 1471-2229-12-23-S8.PPTX]

## Slide 1
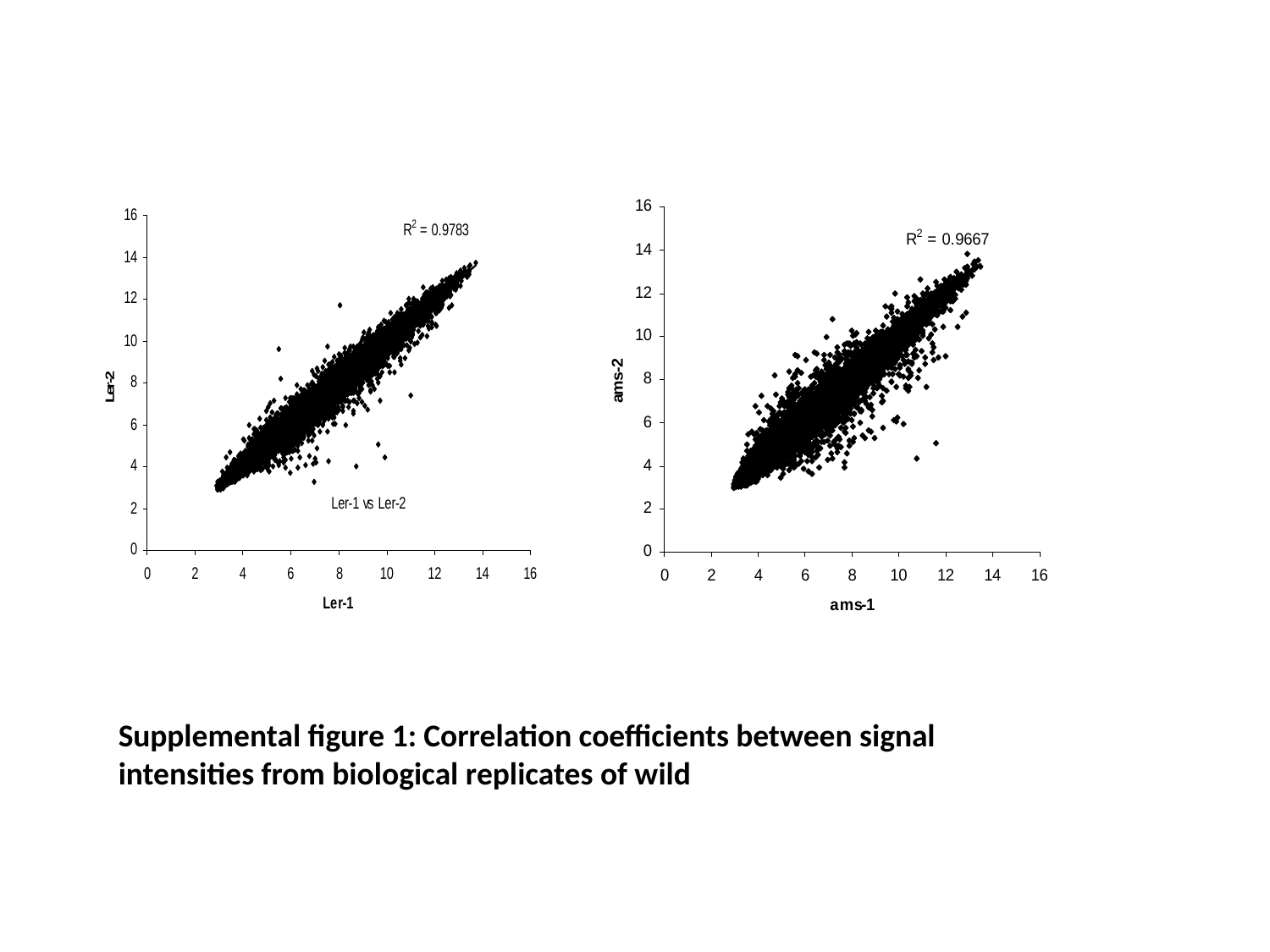

Supplemental figure 1: Correlation coefficients between signal intensities from biological replicates of wild

## Slide 2
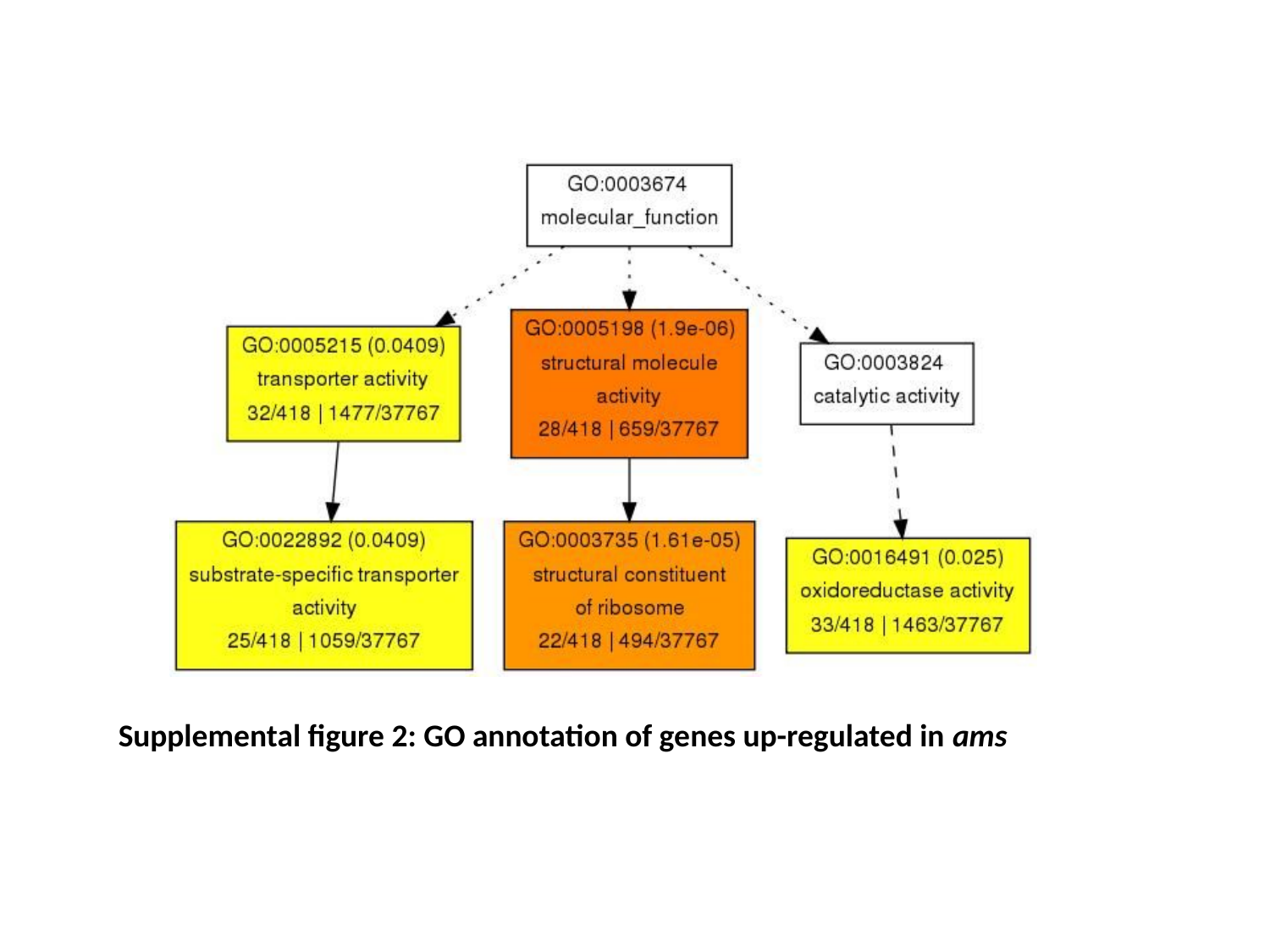

Supplemental figure 2: GO annotation of genes up-regulated in ams

## Slide 3
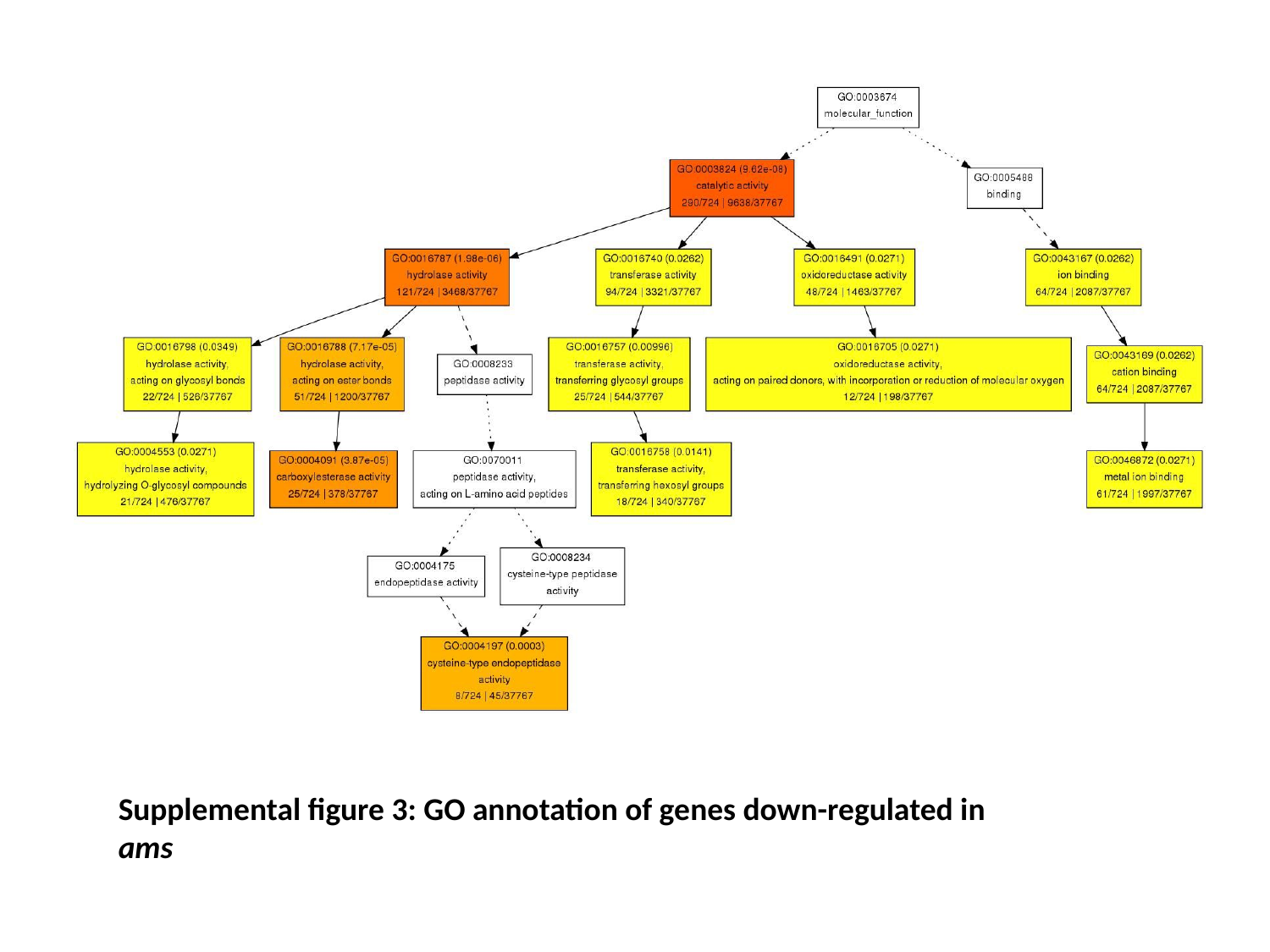

Supplemental figure 3: GO annotation of genes down-regulated in ams

## Slide 4
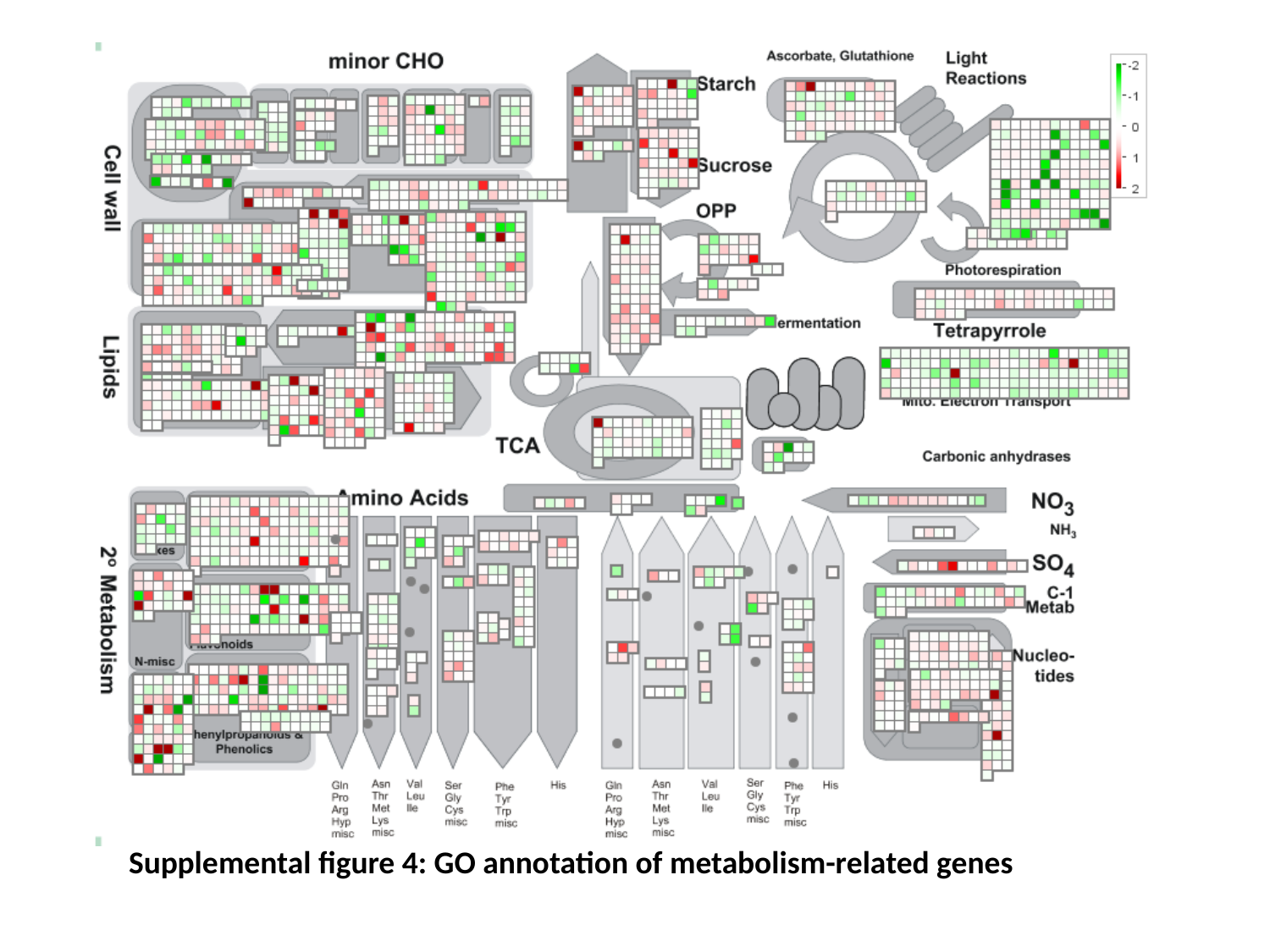

Supplemental figure 4: GO annotation of metabolism-related genes

## Slide 5
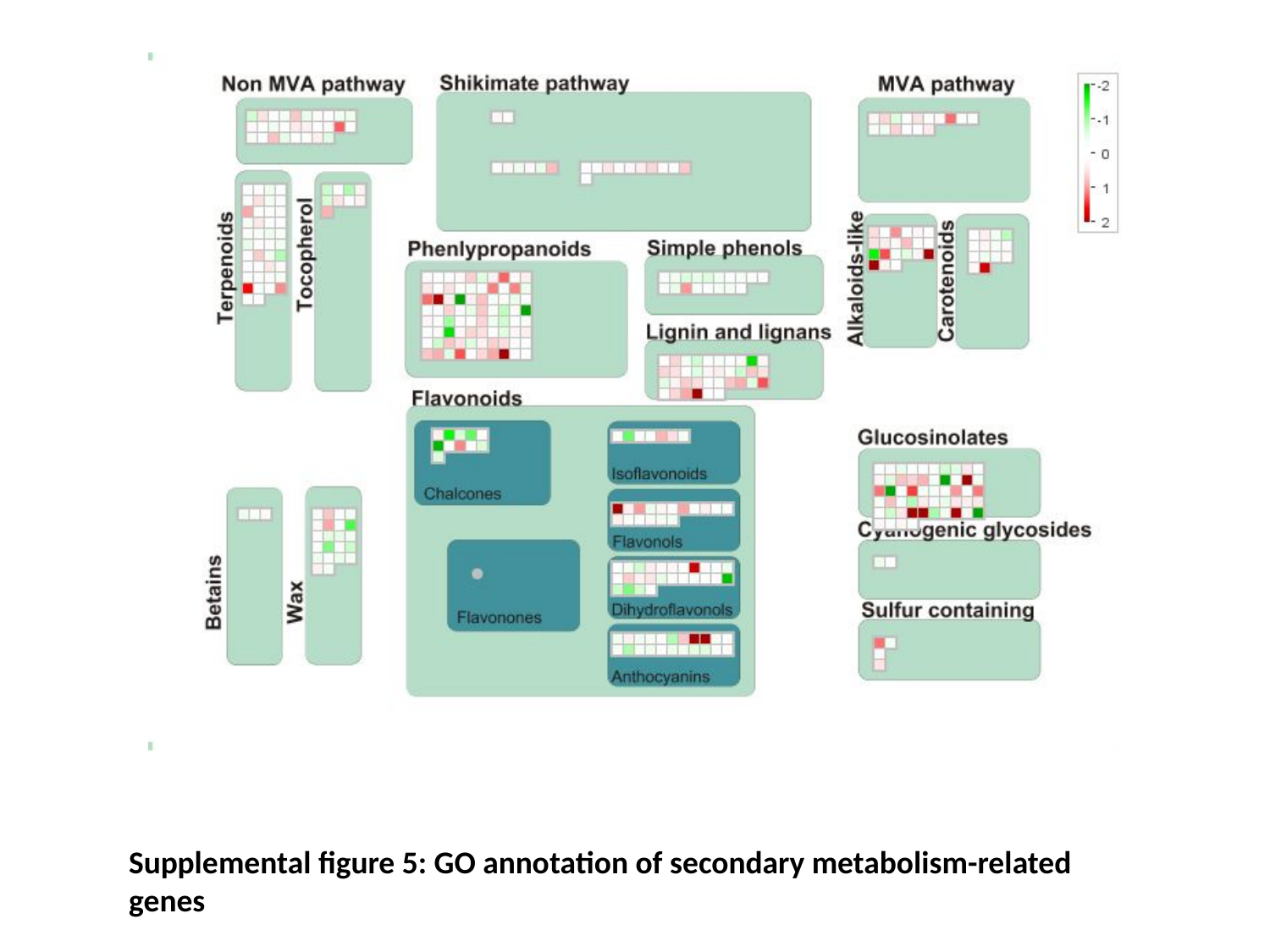

Supplemental figure 5: GO annotation of secondary metabolism-related genes

## Slide 6
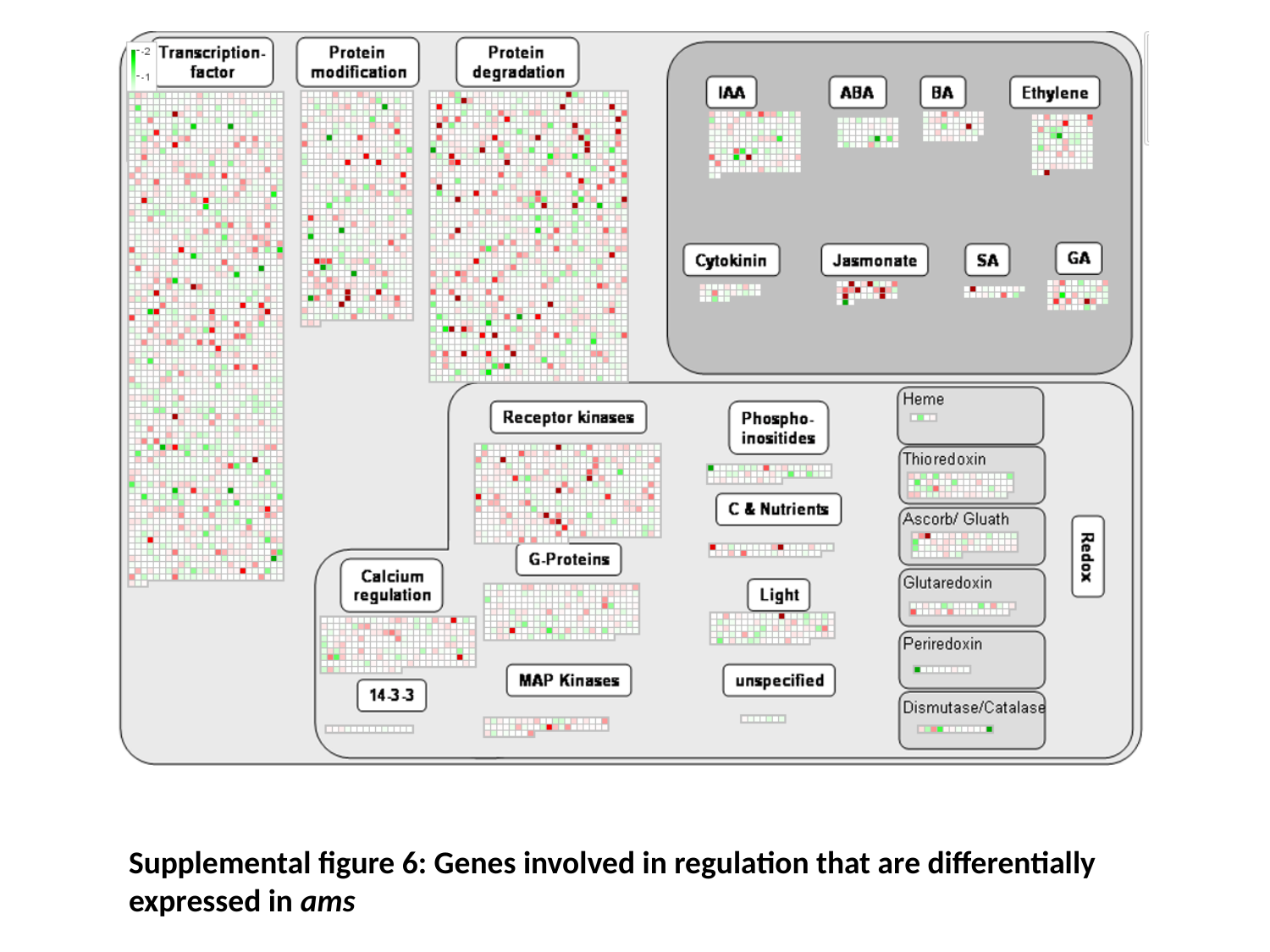

Supplemental figure 6: Genes involved in regulation that are differentially expressed in ams

## Slide 7
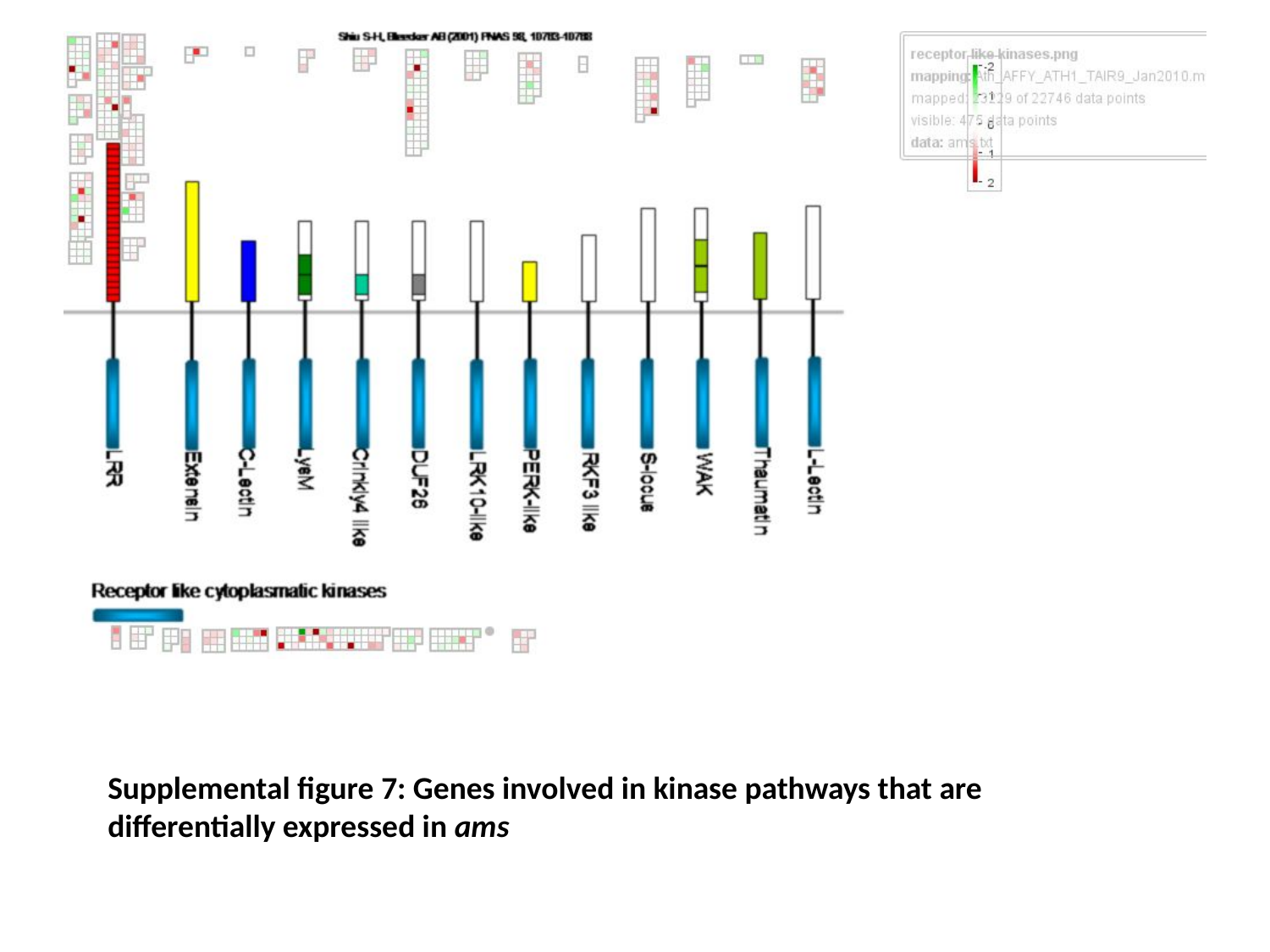

Supplemental figure 7: Genes involved in kinase pathways that are differentially expressed in ams

## Slide 8
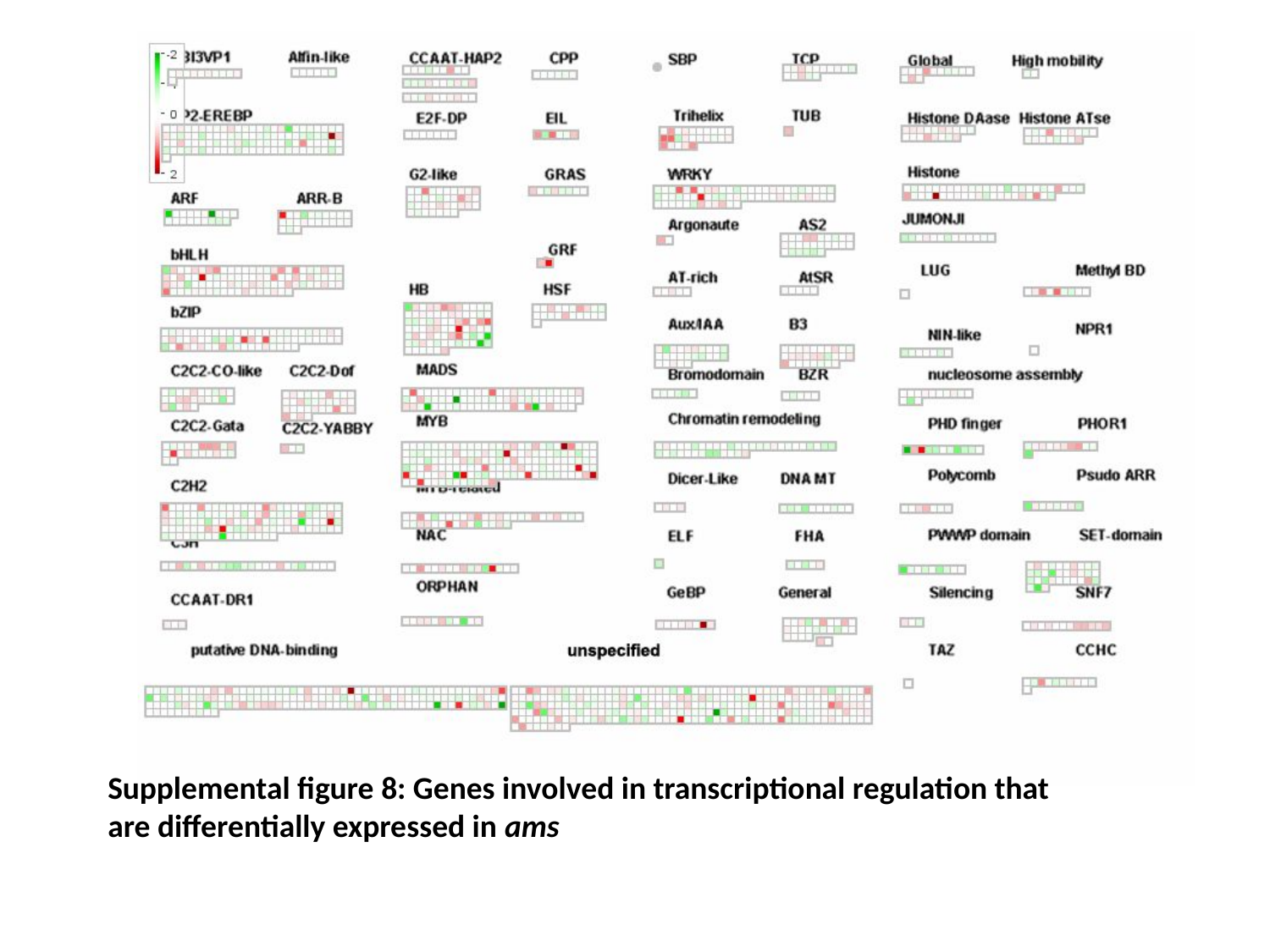

Supplemental figure 8: Genes involved in transcriptional regulation that are differentially expressed in ams

## Slide 9
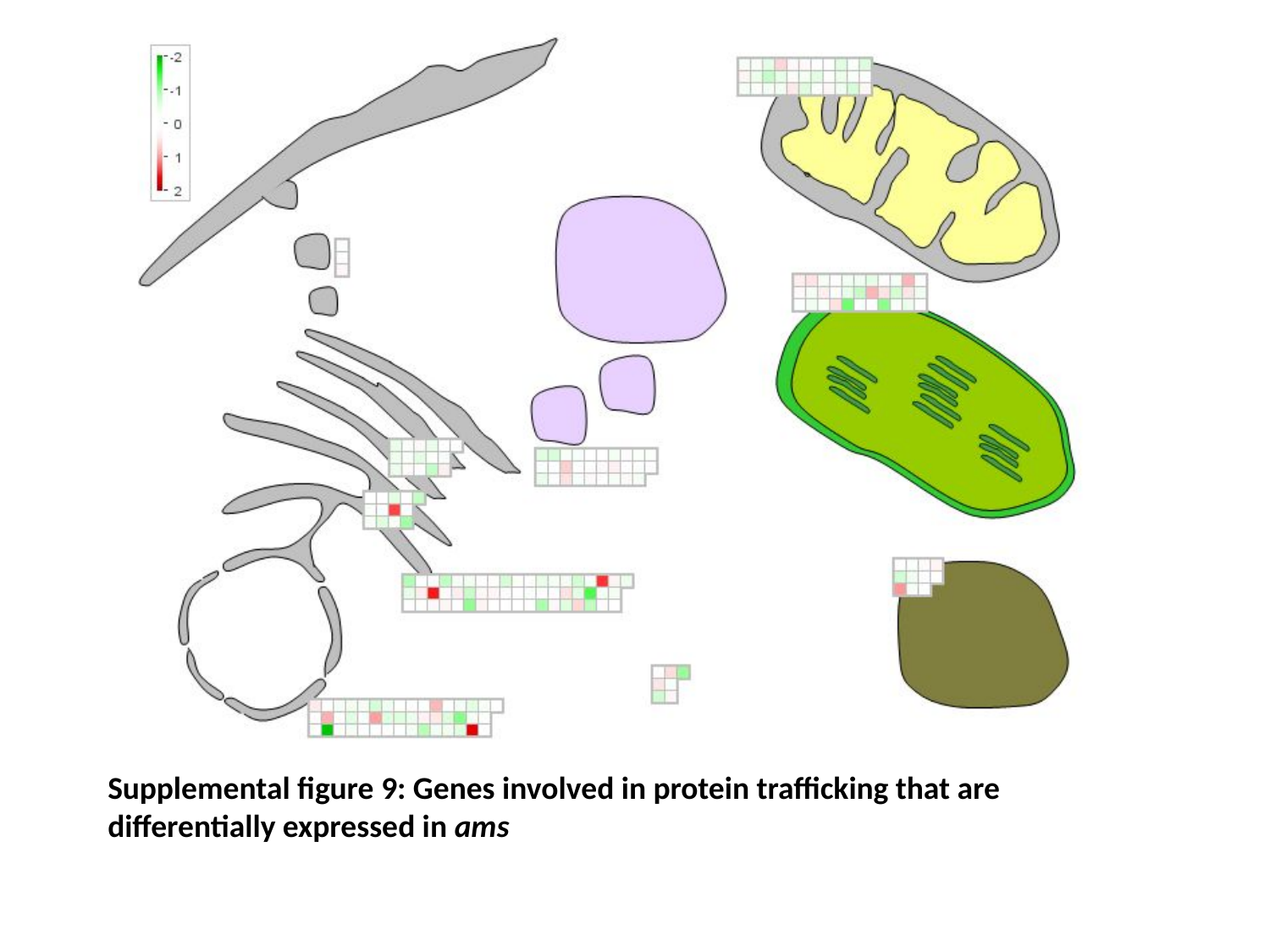

Supplemental figure 9: Genes involved in protein trafficking that are differentially expressed in ams

## Slide 10
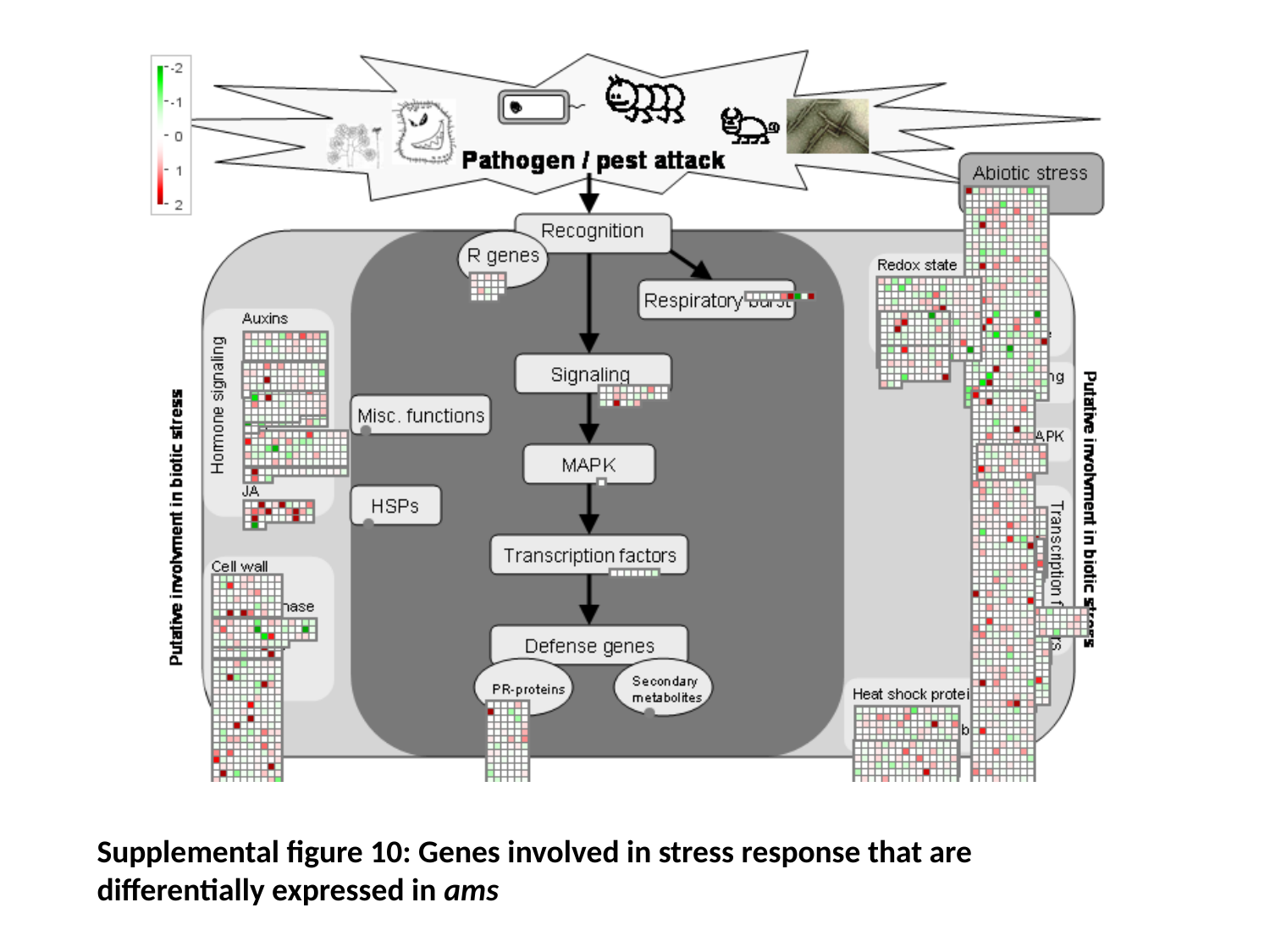

Supplemental figure 10: Genes involved in stress response that are differentially expressed in ams

## Slide 11
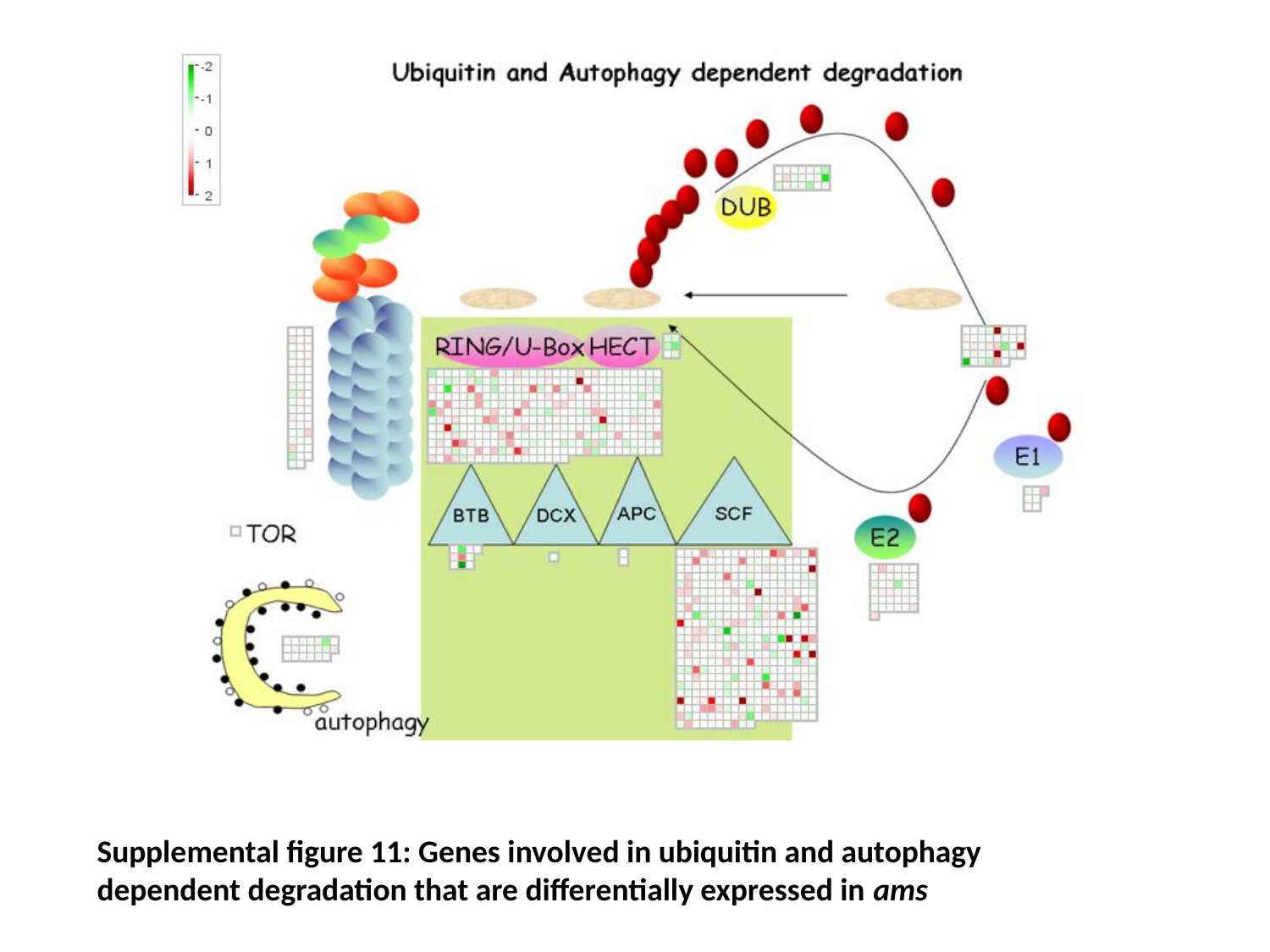

Supplemental figure 11: Genes involved in ubiquitin and autophagy dependent degradation that are differentially expressed in ams

## Slide 12
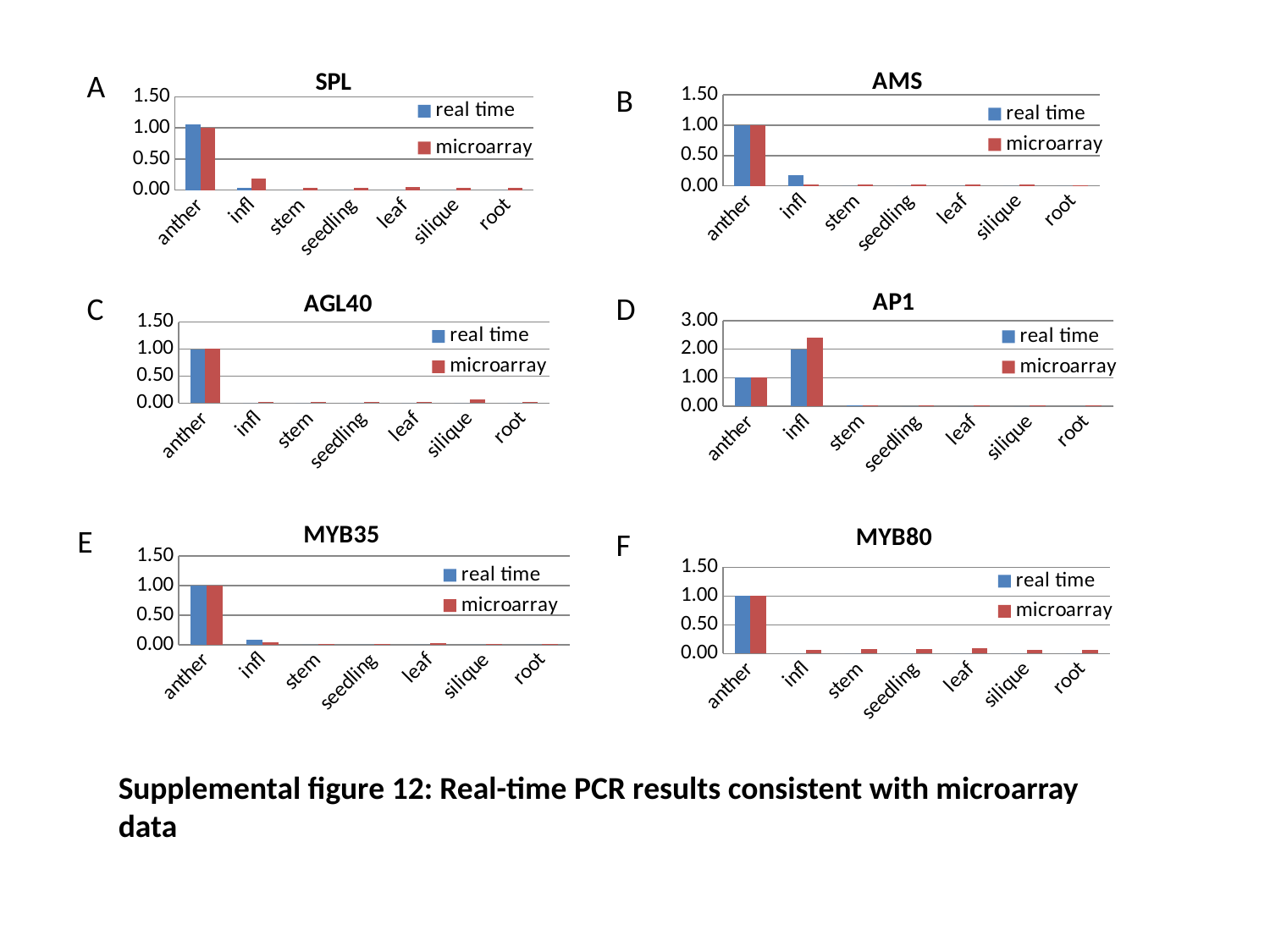

### Chart: AMS
| Category | real time | microarray |
|---|---|---|
| anther | 1.0 | 0.9987392385040926 |
| infl | 0.17 | 0.019955957416814798 |
| stem | 0.0 | 0.016194798286443605 |
| seedling | 0.0 | 0.015718020619095382 |
| leaf | 0.0 | 0.018243437387130225 |
| silique | 0.0 | 0.01649844473672782 |
| root | 0.0 | 0.014918180955918353 |A
### Chart: SPL
| Category | real time | microarray |
|---|---|---|
| anther | 1.05 | 0.9997265879536277 |
| infl | 0.04 | 0.18091413473301732 |
| stem | 0.0 | 0.040650812767970855 |
| seedling | 0.0 | 0.040464257124222386 |
| leaf | 0.0 | 0.04569426343174485 |
| silique | 0.0 | 0.040333362444810124 |
| root | 0.0 | 0.029247118462979386 |B
### Chart: AGL40
| Category | real time | microarray |
|---|---|---|
| anther | 1.0 | 1.0023882738528112 |
| infl | 0.0 | 0.020224298470131317 |
| stem | 0.0 | 0.021167232032762554 |
| seedling | 0.0 | 0.020015168546702853 |
| leaf | 0.0 | 0.02328996605665693 |
| silique | 0.0 | 0.06499009813895584 |
| root | 0.0 | 0.018415467184289096 |
### Chart: AP1
| Category | real time | microarray |
|---|---|---|
| anther | 1.01 | 1.0026780410383744 |
| infl | 2.0 | 2.400898993953609 |
| stem | 0.03 | 0.03407262084680895 |
| seedling | 0.0 | 0.025112373152658237 |
| leaf | 0.0 | 0.04141243345426918 |
| silique | 0.0 | 0.03588428298171247 |
| root | 0.0 | 0.028786955949683488 |C
D
### Chart: MYB35
| Category | real time | microarray |
|---|---|---|
| anther | 1.0 | 0.997663090287521 |
| infl | 0.09 | 0.036732284991061744 |
| stem | 0.0 | 0.018628105781422887 |
| seedling | 0.0 | 0.016588051637413673 |
| leaf | 0.0 | 0.023313853116009184 |
| silique | 0.0 | 0.019346614966118787 |
| root | 0.0 | 0.015897535569488856 |
### Chart: MYB80
| Category | real time | microarray |
|---|---|---|
| anther | 1.0 | 1.000122911576045 |
| infl | 0.0 | 0.06763614588985234 |
| stem | 0.0 | 0.07130703383584559 |
| seedling | 0.0 | 0.07246982962452178 |
| leaf | 0.0 | 0.0902043966500494 |
| silique | 0.0 | 0.06974426048960715 |
| root | 0.0 | 0.060277198415796374 |E
F
Supplemental figure 12: Real-time PCR results consistent with microarray data
